# Enhancing the Cohesion and Influence of Minority Opinions Through Clustering: A Social Network Experiment

**DOI:** 10.1002/pchj.70051

**Published:** 2025-09-21

**Authors:** Baizhou Wu, Jun Liu, Ying Li, Chenran Shen‐Zhang, Shenghua Luan

**Affiliations:** ^1^ Chinese Academy of Sciences State Key Laboratory of Cognitive Science and Mental Health Beijing China; ^2^ Department of Psychology University of Chinese Academy of Sciences Beijing China

**Keywords:** filter bubble, minority influence, public opinion, social influence, social network

## Abstract

Minority opinions can be of crucial importance to the diversity, productivity, and harmony of a group, but are often left unattended and unheard. Previous methods that tried to enhance minority influence are usually overly forceful and low on ecological validity. To overcome these pitfalls, we proposed a new intervention method called *minority clustering* and examined its effects with a social network experiment (*N* = 456). Minority clustering was implemented by increasing the network connections among participants with initial opinions that deviated from the mainstream opinion and forming an opinion cluster among these minority members. Our results show that minority clustering significantly slowed down the rate at which minority members shifted toward majority opinions, thereby sustaining minority cohesion, and moved majority members closer to minority opinions, thus enhancing minority influence. An additional *filter bubble* intervention, through which all members of a network were exposed to neighbors with similar opinions to their own, further strengthened minority cohesion but weakened minority influence. Minority clustering is an unobtrusive intervention that does not need overt cooperations of network members and can be implemented easily in social media platforms. The working mechanisms of minority clustering and its effects on group opinion formation are further discussed.

## 1 Introduction

How public opinions form and evolve has profound implications for human collective behavior and decisions (Bail et al. 2018). They can shape the courses of social events in the short term, inform policy decisions in the intermediate term, and even alter a nation's development trajectory over the long term (Abid and Harrigan 2020). Since Asch's (1951) seminal work, extensive research has shown that individual judgments and decisions are subject to social influences (Behrens et al. 2008), which can be *informational*, when people rely on others for more accurate input (Lorenz et al. 2011); *normative*, when individuals conform to gain social approval (Deutsch and Gerard 1955); or *imitative*, when people follow successful others to pursue their own success (Bandura 1977).

Social influence often unfolds in group settings, where interpersonal interactions create dynamics beyond the individual level (Wu et al. 2020). In recent years, the rise of the Internet and social media has dramatically reshaped these dynamics (Tump et al. 2020). Algorithmic filtering on social media platforms leads to *filter bubbles*, exposing users only to selected content (Flaxman et al. 2016; Nguyen et al. 2014; Pariser 2011), while users tend to engage with and accept information that aligns with their preexisting beliefs while ignoring or rejecting contradictory information, resulting in *echo chambers* (Bakshy et al. 2015; Garrett 2009; Sunstein 2001, 2018). These phenomena contribute to biased information exposure and heightened opinion polarization, shaped jointly by network structures and information‐processing biases of group members.

According to Latané's (1981) social impact theory, social influence is modulated by source status and proximity. Building on this, network‐based models use graph theory to formalize social structures and quantify influence (Borgatti et al. 2009). A foundational example is DeGroot's (1974) model, which conceptualizes opinion updating as a weighted average of peers' views. Since then, mechanisms of group opinion formation have been explored through simulations (Barkoczi and Galesic 2016; Burt 1992; Zarei et al. 2024), behavioral experiments (Becker et al. 2017; Rand et al. 2011), and hybrid studies (Jayles et al. 2017).

Social network experiments offer a controlled, quantitative framework for examining group decision making and opinion dynamics. In a network experiment, participants typically answer the same question across multiple rounds and adjust their responses based on others' inputs, mimicking how real‐world interactions unfold in group contexts. Group opinions are often represented by measures such as the mean, median, or geometric mean of individual opinions (Kao et al. 2018), which capture central tendencies but risk overlooking minority perspectives. As Salganik and Watts (2008) noted, even accurate or well‐reasoned views can be eclipsed by dominant majorities. In political contexts, Downs (1957) argued that voters align with parties based on ideological identification rather than policy specifics, highlighting the role of party loyalty. Groups composed of ideologically divergent members—heterogeneous groups—benefit from internal competition and compromise, which can foster diverse viewpoints and enhance group performance (Page 2007). Maintaining diversity and amplifying minority voices is thus essential for creative, well‐informed collective decisions.

The majority members of a group have numerical advantages and richer interpersonal connections, making their opinions more likely to spread and dominate within network interactions. However, the minority can still exert significant influence under certain conditions. Recent social movements, such as feminism, environmentalism, and gender identity, demonstrate that minority viewpoints can gain traction and significantly shape mainstream public opinions. These movements often start as niche perspectives among a small group of adherents before expanding into global trends.

The role of minorities in opinion shifts has generally been underestimated, yet research has shown both explicit and implicit ways through which minorities can affect the formation of group opinions (Maass and Clark III 1984; Moscovici 1980; Nemeth 1986; Wood et al. 1994). For instance, Moscovici et al. (1969) found that when minority members maintain consistent opinions, they can exert a disproportionate influence on the majority. Other studies show that even a small but unwavering minority group dispersed within a network can quickly reverse mainstream opinions (Mobilia 2003; Xie et al. 2011; Yildiz et al. 2013).

Although these studies demonstrate the potential power of the minority, their findings have been constrained by methodological limitations. Besides drawing conclusions on simple experimental paradigms and small sample sizes, many of these studies artificially amplified minority influence by introducing unrealistic and extreme experimental manipulations, such as requiring minority participants to strictly comply with the experimenter's instructions or to maintain unwavering positions throughout the task (Bonacich 1987; Granovetter 1978). Such manipulations do not align with real‐world settings and pose serious challenges to the external and ecological validities of study findings.

In recent years, some researchers have investigated group opinion formation through online social network tasks. For instance, Centola (2010) conducted a controlled experiment on behavior diffusion in online health communities, finding that clustered‐lattice networks, in which nodes form tightly connected clusters, were more conducive to the spread of socially reinforced behaviors than random networks. This effect occurs because redundant connections within clusters expose individuals to multiple reinforcing signals, making them more likely to adopt new behaviors. In a more tightly controlled group image‐naming experiment, Centola et al. (2018) found that established social conventions in groups of 20–30 people could be overturned by a committed minority whose size was approximately 20% of the group. In general, social network experiments allow researchers to examine large‐scale group processes while precisely controlling the flow and reception of social information among participants.

The purpose of the present study is threefold: (a) to examine minority influence on group opinion formation in a more relevant and ecologically valid setting; (b) to test the effect of a less intrusive and more naturalistic manipulation of minority influence; and (c) to investigate how the cohesion of minority members and their influence on majority members change dynamically through multiple interactions. To these ends, we conducted a social network experiment in which participants saw others' opinions and updated their own opinions repeatedly in real time, and introduced a novel manipulation of minority influence: We first identified isolated individuals within the network whose opinions deviated from the mainstream but were aligned with each other, and then reinforced the network connections among these individuals. This design increases the likelihood that each minority member is exposed to others with similar views, thereby forming a connected minority cluster. Unlike previous studies, participants in our experiment were free to process social information and respond autonomously, and none of them were instructed to covertly cooperate with the experimenter as stubborn opinion holders.

We also investigated the effects of filter bubbles by feeding all individuals in a network with social viewpoints similar to theirs. While filter bubbles may strengthen opinion cohesion among minority members, they do not necessarily yield the same effect among majority members. This asymmetry arises because majority opinions are often more dispersed, making it more difficult for majority individuals residing in different bubbles to converge on the same position. Moreover, we asked participants to express their opinions toward ongoing social issues. This helps situate opinion exchanges in a more subjectively relevant and ecological context, and allows us to test the effects of our manipulations in a tightly controlled yet realistic environment.

## 2 Methods

### 2.1 Participants

A total of 456 participants (*M*_Age_ = 22.15, SD_Age_ = 1.86, # of females = 370) took part in and completed the experiment. All participants were students enrolled at a university in Northeastern China and were recruited through advertisements in social media. They were compensated with a fixed participation fee of ¥10 (roughly $1.40). All participants signed written informed consent forms before participating in the study.

### 2.2 Procedure and Materials

The experiment was conducted on an Internet‐based platform that can simulate critical elements of real‐world social networks and allows participants to engage in real‐time interactions in a laboratory setting. In the experiment, each participant was randomly assigned to a network group. Once all participants of a group were available, they were asked to access to the experiment platform via a laptop or a tablet and complete their responses to questions related to three social issues in a network together. In each session, participants were first asked to read an instruction passage and complete two practice questions to familiarize themselves with the task, and then completed the three targeted questions. The entire experiment lasted approximately 20 min, and the three targeted questions are as follows.

Q1: “What do you think is the main reason that people purchase Huawei products?” For this question, “1” indicates that it is entirely based on product quality, and “99” indicates that it is entirely based on patriotism. Q2: “What do you think is the primary cause of the US‐China trade war?” For this question, “1” indicates that it is entirely caused by economic factors, and “99” indicates that it is entirely caused by political factors. Q3: “Who do you believe should bear the primary responsibility for the Palestine‐Israel conflict?” For this question, “1” indicates Palestine and “99” indicates Israel.

**FIGURE 1:**
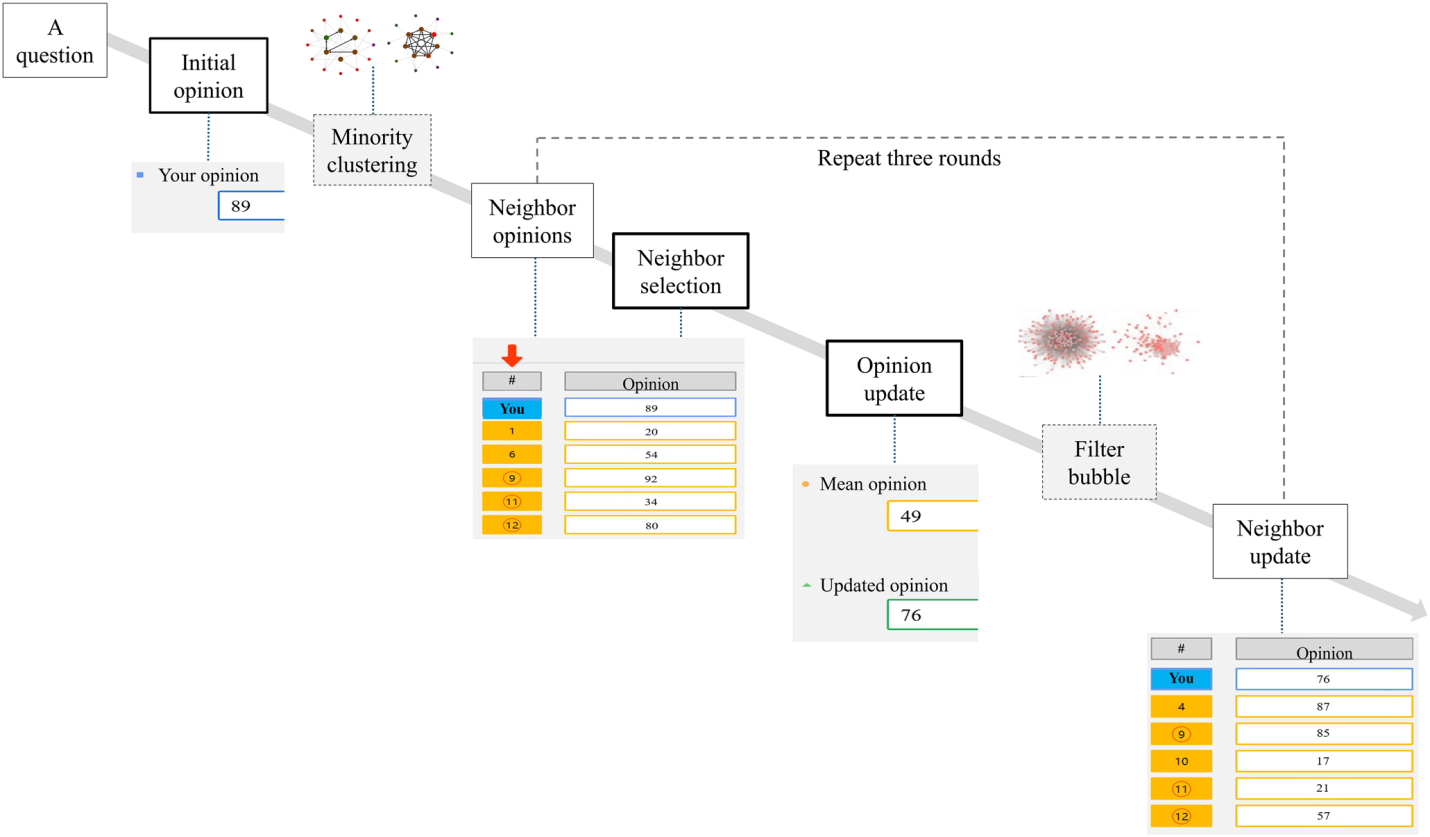
Experimental procedure. The thin and bold solid‐lined frames represent the viewing and the response stages, respectively, in an experiment trial; graphs associated with these frames are screenshots of a trial. The dashed frames denote two manipulations of the experiment that were controlled by the experimenter and performed in the backend of the experiment program; graphs associated with these frames show stylized effects of these manipulations. Details of the two manipulations, minority clustering and filter bubble, are described in the section “Independent Variables.”

For each question, the question itself was first presented on the screen (Figure 1). Participants responded by keying in an integer between 1 and 99 to express their initial opinion. They were then shown the opinions of five network neighbors, as well as the mean of these opinions, and asked to update their own opinion. After that, participants could choose to retain 0–5 of the neighbors whose opinions they just saw. The discarded neighbors would be subsequently replaced by other participants in the network. Opinions of these new neighbors, as well as the updated opinions of the retained neighbors, were then shown to the participants, who were asked once again to update their opinion. For each question, the process was repeated for three rounds, and a total of four responses from each participant were recorded: an initial response and three updated ones.

### 2.3 Experimental Design

A 2 (minority clustering: yes and no) × 2 (filter bubbles: yes and no) between‐subjects design was used. Each condition included four separate network groups with an average of 28 participants in each, and Table S1 reports the number of participants in each network group.

### 2.4 Independent Variables

Minority clustering was designed to connect isolated minority members by altering the network relationship among these members. In the no‐clustering condition, all participants' neighbors were randomly picked in the first round. If some neighbors were discarded in the next two rounds, they were also replaced by random members in the network. In the clustering condition, the assignment and replacement of neighbors for the majority members followed the same procedures as in the no‐clustering condition; however, this was not the case for the minority members.

Specifically, in the first round, three of the five neighbors shown to a minority member were also minority members (randomly picked), while the other two were randomly selected majority members. In the second round, a discarded minority or majority neighbor would be replaced by a random member of the same type. In the third round, the replacement of neighbors for minority members followed the same random procedure as for majority members. Participants were not informed of these neighbor assignment and replacement processes.

We used a minority search algorithm (Table 1) to identify seven participants as the minority members in a group of approximately 28 participants (≈25%). This proportion exceeds the critical mass proposed by Centola et al. (2018), which found that a committed minority could overturn an established social convention when it constituted around 20% of the total group size. Minority and majority members were determined based on participants' initial opinions, and for each question, a participant's type (i.e., minority or majority) was fixed throughout all rounds.

**TABLE 1:** Steps of the minority search algorithm.

Step	Procedure
1	Sort all participants' initial opinions in ascending orders and select the first seven as members of a candidate minority.
2	Calculate the absolute distance between median of this group's opinions and that of the entire network group.
3	Compute the kurtosis of the opinion distribution, a measure of distribution concentration, of the candidate group, and multiply it by the distance score in Step 2 to create a minority index. A minority group with a higher index score is a group that is either more internally consistent (i.e., more clustered) or whose overall opinion deviates more from the mainstream opinion (i.e., more a minority), or both.
4	Change the candidate minority group to include the second to eighth participants as ordered in Step 1, and calculate the minority index of this group.
5	Repeat Steps 1–4 with the next seven participants in the order, and choose the candidate group with the highest minority index as the minority group that would be subject to clustering manipulation.

Minority members are positioned at the periphery of a network. In the absence of intervention, isolated minority members can be highly susceptible to majority assimilation. With the clustering manipulation, however, minority members are placed in an environment where they interact primarily with individuals holding similar opinions while remaining some distance away from the majority. In this environment, minority members have a higher chance of maintaining their opinions whether they are socially influenced or not.

With respect to filter bubbles, in the no‐bubble condition, neighbors of a participant were randomly selected from the network in each round. In the bubble condition, neighbors were selected based on the similarity between their opinions and a participant's current opinion, and the higher the similarity, the more likely the selection. Specifically, opinion similarity was determined with Equation (1) in each round.

(1) $${\text{Similarity}}_{i\to n}={\left(1-\frac{\left|x_{n}-x_{i}\right|}{1+\left|x_{\max}-x_{\min}\right|}\right)}^{\mathrm{st}}$$

The left part of the equation is the opinion similarity between a candidate participant *i* and participant *n*, who has undetermined neighbors. In the right part, $x_{n}$ is the opinion of participant *n*; $x_{i}$ is the opinion of a participant who is yet participant *n*'s neighbor; $x_{\max}$ and $x_{\min}$ represent the maximum and minimum opinion values among all network members in the present round; and *st* is the strength coefficient that can take any value between 0, making all candidate neighbors equally similar to participant *n*, to infinite. After the calculation of opinion similarity, the selection probability of a candidate neighbor $P_{i}$ is then determined with Equation (2), in which $S_{i}$ denotes opinion similarity between candidate *i* and participant *n*.

(2) $$P_{i}=\frac{S_{i}}{\sum_{i}S_{i}}$$

In the experiment, the similarity strength coefficient *st* was set to 20 in the bubble condition. Based on our simulation explorations, this magnitude can greatly amplify differences in similarity score and render candidate participants holding more similar opinions much higher probabilities of being selected as neighbors than other participants.

### 2.5 Key Dependent Variables

To examine how participants updated their opinions under the co‐influences of minority and majority members, we constructed two indices. For each participant, the *minority deviation score* at each round is calculated with Equation (3).

(3) $$D_{i,r}^{\min}=\left|\frac{E_{i,r}-M_{0}^{\min}}{M_{0}^{\mathrm{maj}}-M_{0}^{\min}}\right|-\left|\frac{E_{i,0}-M_{0}^{\min}}{M_{0}^{\mathrm{maj}}-M_{0}^{\min}}\right|$$

In the equation, *i* denotes a participant and *r* the round number; $E_{i,r}$ represents participant *i*'s response in round *r*; $M_{0}^{\min}$ and $M_{0}^{\mathrm{maj}}$ are the initial median opinions of the minority group and the majority group before network interaction, respectively. The first term on the right‐hand side represents a standardized distance between a participant's current opinion and the minority opinion median. When $E_{i,r}=M_{0}^{\min}$, that distance is 0; when $E_{i,r}=M_{0}^{\mathrm{maj}}$, it equals to 1. If the denominator is zero (i.e., $M_{0}^{\mathrm{maj}}=M_{0}^{\min}$), the distances to both groups are equal, and the deviation score is assigned a default value of 0.50. The second term represents the distance between a participant's initial opinion from the minority opinion median. Overall, a larger value of $D_{i,r}^{\min}$ indicates a greater shift away from the initial minority position.

Analogously, the *majority deviation score* is calculated with Equation (4), and a larger value of $D_{i,r}^{\mathrm{maj}}$ indicates a greater shift away from the initial majority position.

(4) $$D_{i,r}^{\mathrm{maj}}=\left|\frac{E_{i,r}-M_{0}^{\mathrm{maj}}}{M_{0}^{\mathrm{maj}}-M_{0}^{\min}}\right|-\left|\frac{E_{i,0}-M_{0}^{\mathrm{maj}}}{M_{0}^{\mathrm{maj}}-M_{0}^{\min}}\right|$$

For participants whose opinions fall between $M_{0}^{\min}$ and $M_{0}^{\mathrm{maj}}$, an increase in one deviation score implies a decrease in the other. However, for those whose opinions lie outside this range—that is, lower than the minority median or higher than the majority median—both scores may increase or decrease simultaneously. In such cases, neither score alone sufficiently captures the direction of influence. Therefore, an *opinion leaning index* is further calculated to capture the overall shift of a participant’ opinion (Equation 5).

(5) $${\Delta D}_{i,r}=D_{i,r}^{\mathrm{maj}}-D_{i,r}^{\min}$$

This index is the difference between the majority deviation score and the minority deviation score. A positive value indicates that relative to their initial position, a participant's opinion has deviated more from the majority opinion (and shifted closer to the minority opinion) in round *r*, and a negative value has the opposite indication. Intuitively, this leaning index can be understood as the result of a tug‐of‐war between the majority influence and the minority influence: If a participant's opinion deviates more from the majority (the first term in Equation 5) than from the minority (the second term), the minority is inferred to have exerted comparatively more influence than the majority, and a reverse inference is drawn otherwise.

Lastly, to understand the impact of minority members on group opinions, we analyzed two aspects separately: (1) *minority cohesion*, which is the ability of minority members to maintain their initial stance; and (2) *minority influence*, which is minority members' ability to alter the opinions of majority members. Both were quantified with the opinion leaning index ${\Delta D}_{i,r}$ but with a different analysis target: For minority cohesion, the target was the minority members and the index was calculated based on responses from these members only; for minority influence, it was the majority members.

## 3 Results

**FIGURE 2:**
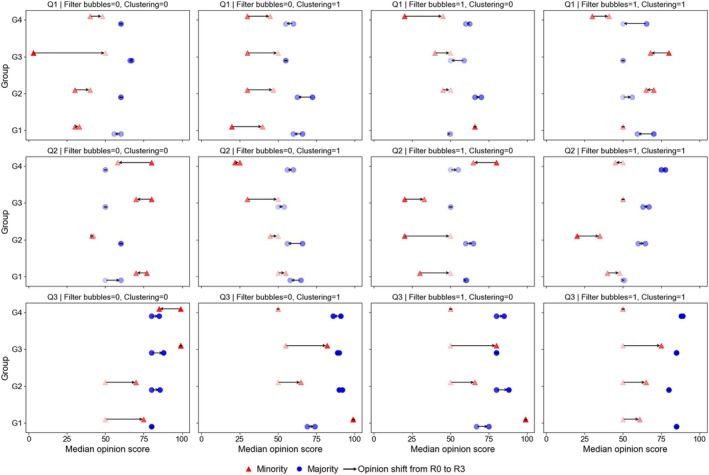
Opinion shifts of the minority and the majority members. Each panel presents results of four participant groups (i.e., G1 to G4) in a given experimental condition and for a specific question (i.e., Q1, Q2, and Q3). “Filter bubbles” and “Clustering” refer to the two experimental manipulations, with a value of 1 indicating the presence of a manipulation and 0 the absence. Each group is divided into two subgroups: The minority (red triangle) and the majority (blue circle), and each symbol denotes the median opinion of their respective subgroup. Darker colors represent more extreme opinions (closer to 1 or 99), while lighter colors indicate more moderate opinions (closer to 50). Arrows indicate how the median opinion within the same subgroup shifted from Round 0 (R0) to Round 3 (R3).

Part II of the SIs shows the detailed opinion distributions of all 16 participant groups in each round and for each question, while Figure 2 illustrates the opinion shifts from Round 0 (before network interaction) to Round 3 (last round) for the minority and the majority members of each participant group. Three general patterns can be observed from the figure. First, at Round 0 and for each question, the median majority opinions were quite similar across all 16 groups, and there were clear separations between the majority and the minority median opinions in all cases. Second, opinions of the two subgroups tended to move toward each other after network interaction, with the magnitudes of the shifts generally larger for minority members than for majority members. Third, for both the majority and the minority members, the magnitudes of opinion shift apparently differed across experimental conditions. However, it is difficult to pin down the exact effects of the two experimental manipulations from these first‐level descriptive results.

**FIGURE 3:**
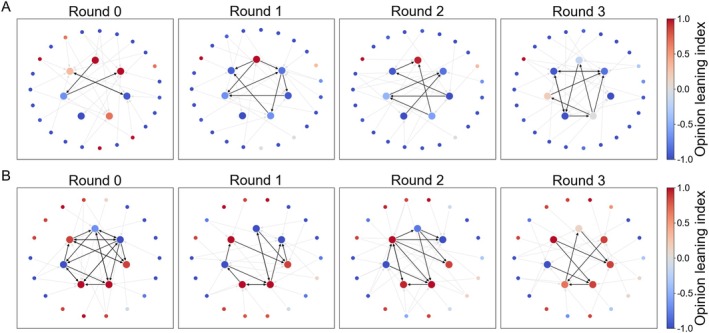
Dynamic changes of member opinions of two specific participant groups. Both groups responded on Q2 (i.e., US–China trade war) in the condition of no filter bubbles. Panel A shows results of a group without the clustering manipulation, while Panel B shows those of a group with the clustering manipulation. In each panel, the inner (and larger) nodes represent minority members, and the outer (and smaller) nodes represent majority members. Arrows indicate the presence and directions of social influence in a round. Node colors represent magnitudes of the *unadjusted* opinion leaning index, which means that both the minority and the majority deviation scores were calculated without corrections of their baseline levels at Round 0, thereby preventing the leaning index from degenerating into zero at Round 0. Positive (red) and negative (blue) values of the index indicate opinions closer to the median initial opinion of the minority members and that of the majority members, respectively.

Figure 3 provides a more fine‐grained depiction of how the opinions of members of two specific participant groups changed over the rounds for a specific question, while Part III of the SI shows the dynamic opinion evolutions of all participant groups and for all questions. For the group without the clustering manipulation (Panel A), the minority members were less connected, and over time, their opinions gradually shifted toward the initial median opinion of the majority, to the extent of almost total assimilation. In contrast, for the group with the clustering manipulation (Panel B), the minority members were more connected with each other to start with and remained connected throughout the rounds. At the same time, they managed to not only withhold their initial stances over time, but also turn the opinions of some majority members in the minority direction. Overall, the results of these two participant groups demonstrate the possible effects of the clustering manipulation. To test whether these effects were generally present in the experiment, however, further analyses are needed.

As mentioned previously, the key dependent measure examined in our study is the opinion leaning index (Equation 5). It represents the relative strength of influence of the minority and the majority opinions on a participant's position. A higher value of the index indicates that, relative to their initial positions, participants' opinions were closer to the median of the initial minority opinions and farther away from the median of the initial majority opinions. For participants of a given group, we first calculated the index score for all responses to a specific question, and then tallied the scores separately for responses produced by the minority members and those by the majority members across questions. These scores were the basis for our subsequent analyses of minority cohesion (i.e., the ability of minority members to maintain their stance) and minority influence (i.e., the ability of minority members to shift the opinions of majority members in the minority direction).

**FIGURE 4:**
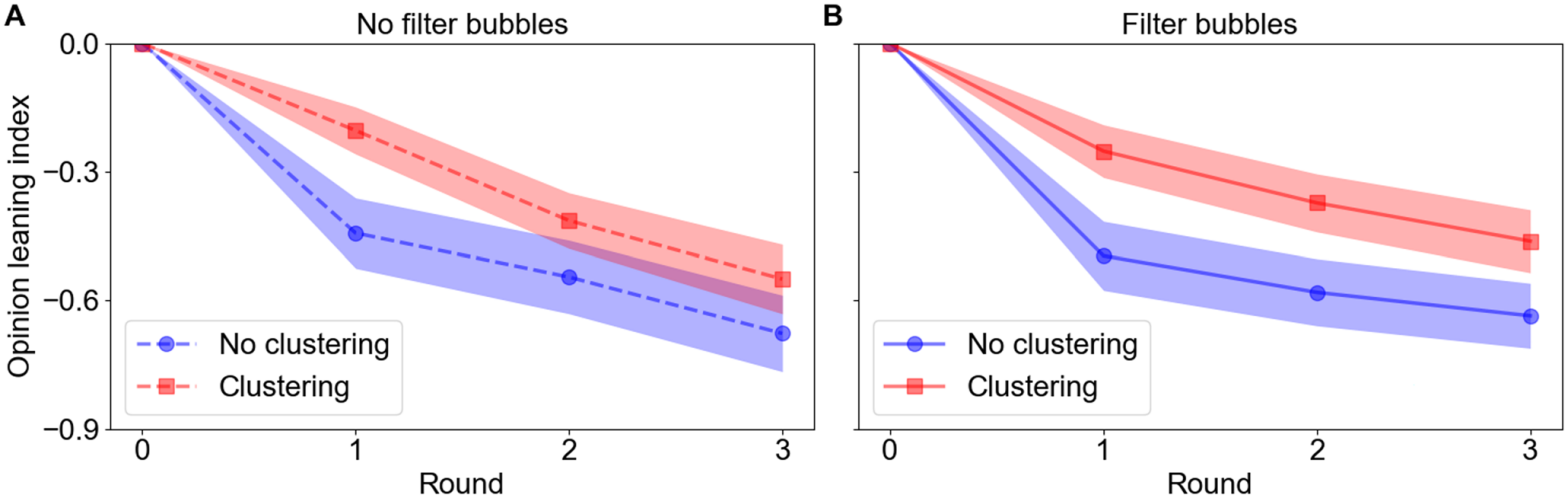
Effects of experimental manipulations on minority cohesion. Each point in the figure represents the mean opinion leaning index of minority members in a round under a certain experimental condition, and bands around the lines indicate standard errors. When a participant was a minority member, we calculated the leaning index of the participant and took the median of the index scores over all situations when that participant was a minority member. We then calculated the mean of the medians over all participants who were ever a minority member in any of the four participant groups in each experimental condition.

Figure 4 shows the results on minority cohesion and illustrates how the opinion leaning index of minority members changed over the rounds in each experimental condition. Across all conditions, minority cohesion declined over time, indicating that minority members increasingly deviated from their initial position and shifted toward the initial majority position, consistent with the general pattern observed in Figure 2. However, compared to the declines in the no‐clustering condition, the declines in minority cohesion were apparently less pronounced in the clustering condition, suggesting that clustering helped sustain minority cohesion over time. Moreover, filter bubbles seemed to slow down the declines of minority cohesion, especially in the latter rounds.

To examine the effects of the experimental manipulations more precisely, we ran a series of mixed‐effects models to analyze data of minority members. Mixed‐effects models are widely applied in psychological research because of their flexibility and suitability to handle complex data (e.g., Barr et al. 2013; Kuznetsova et al. 2017). Each model we ran included the main (and fixed) effects of clustering, filter bubble, and round but with a different combination of interaction effects among the three variables. Table S2 reports the fits—measured by AIC and BIC—of these models, and Table 2 shows the results of the model with the best fit (i.e., the lowest AIC and BIC values). In the model, statistical significances were assessed with the *F*‐tests of fixed effects, and effect sizes with the standardized regression coefficient *β* of each effect.

**TABLE 2:** The mixed‐effects model results on the opinion leaning index of minority members (*n* = 258).

Fixed effects				Standardized regression coefficients					
Variable	df	*F*	*p*	*β*	SE	df	*t*	*p*	95% CI
Clustering	255.00	7.235	0.008	0.332	0.124	255.00	2.690	0.008	[0.089, 0.576]
Bubble	471.80	1.772	0.184	−0.196	0.147	471.80	−1.331	0.184	[−0.484, 0.093]
Round	514.00	108.404	< 0.001	−0.260	0.028	514.00	−9.130	< 0.001	[−0.315, −0.204]
Bubble × Round	514.00	6.755	0.010	0.104	0.040	514.00	2.599	0.010	[0.025, 0.182]

Abbreviations: Bubble = filter bubble; CI = confidence interval; SEM = standard error of the mean.

In terms of main effects, the model shows a significant effect of clustering (i.e., minority members were more coherent in the clustering condition), a significant effect of round (i.e., minority members became less coherent over time), but a nonsignificant effect of filter bubble. That said, the interaction between filter bubble and round was significant, indicating that the filter bubble manipulation did help reduce the decline of minority cohesion but only in the latter rounds. Overall, the modeling results support the observed effects of the experimental manipulations demonstrated in Figure 4.

**FIGURE 5:**
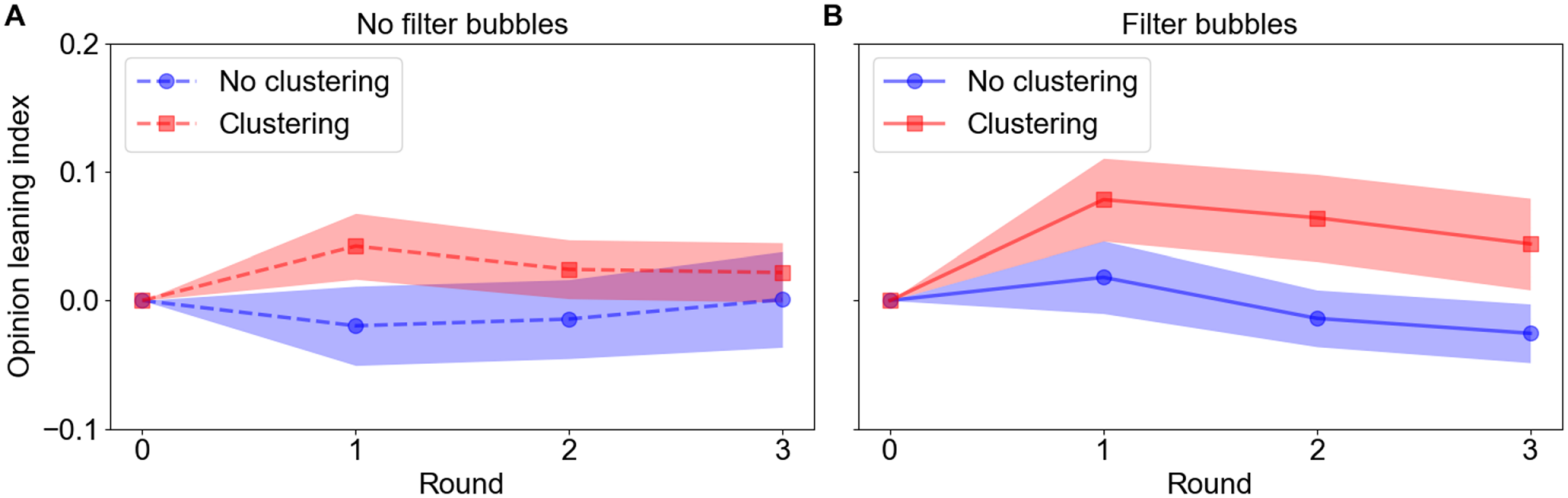
Effects of experimental manipulations on minority influence. Each point in the figure represents the mean opinion leaning index of majority members in a round under a certain experimental condition, and bands around the lines indicate standard errors. The calculation processes were identical to those for minority cohesion, except that the targets were the majority instead of minority members.

Figure 5 shows the results of minority influence on majority members and illustrates how the opinion leaning index of majority members changed over the rounds in each experimental condition. Table 3, meanwhile, reports the results of the best‐fitting mixed‐effects model. There are several notable patterns: (1) the leaning index scores oscillated around 0 in the no‐clustering conditions, implying very little minority influence there, but were positive in the clustering conditions, suggesting the presence of minority influence; (2) the mixed‐effects model shows that similar to its effect on minority cohesion, there was indeed a main effect of clustering on minority influence—that is, the clustering manipulation significantly enhanced minority influence; (3) different from minority cohesion, there was no effect of round on minority influence, indicating that minority influence did not vary much across rounds; and (4) although the main effect of filter bubble was not significant, the interaction between filter bubble and round was; that is, filter bubble boosted minority influence earlier in network interactions but its effect diminished over time. This effect was in the opposite direction of the same interaction for minority cohesion.

**TABLE 3:** The mixed‐effects model results on the opinion leaning index of majority members (*n* = 452).

Fixed effects				Standardized regression coefficients					
Variable	df	*F*	*p*	*β*	SE	df	*t*	*p*	95% CI
Clustering	449.00	4.219	0.041	0.205	0.100	449.00	2.054	0.041	[0.009, 0.401]
Bubble	930.70	2.959	0.086	0.213	0.124	930.70	1.720	0.086	[−0.030, 0.456]
Round	902.00	3.915	0.048	0.000	0.026	902.00	−0.006	0.995	[−0.052, 0.052]
Bubble × Round	902.00	3.881	0.049	−0.072	0.037	902.00	−1.970	0.049	[−0.144, 0.000]

Abbreviations: Bubble = filter bubble; CI = confidence interval; SEM = standard error of the mean.

Recall that we selected seven participants in a network group as minority members following the procedure described in Table 1 and treated all other participants as majority members. This classification could lead to a sizable opinion heterogeneity among the majority members, and a subset of them might hold initial opinions closer to the minority than to the other majority members, thus subjecting themselves more to minority influence. To test the robustness of the minority influence results, we ran the same mixed‐effects model reported in Table 3 but without those “minority‐friendly” members of the majority. Detailed results of this analysis can be found in Part IV of the SI, and they show a significant main effect of clustering and a significant interaction effect between filter bubble and round, replicating the key results in Table 3 and indicating that the heterogeneity of the majority members had little impact on the minority influence results.

Altogether, our results show that the minority clustering manipulation increased the cohesion of minority members' opinions and slowed down their assimilation to the mainstream majority opinions. The manipulation also enhanced the influence of minority members on majority members, able to move the opinions of the latter in the minority direction. Meanwhile, the filter bubble manipulation implemented in our study, with which all participants in a network were more likely exposed to opinions similar to theirs, facilitated minority cohesion but weakened minority influence over time.

## 4 Discussion

The majority usually dictates the formation of group opinions. Previous research seeking to enhance minority influence has adopted relatively explicit and rigid methods, such as creating stubborn minorities by requiring minority members to remain unyielding in their views and make no compromises (e.g., Moscovici et al. 1969; Mobilia 2003; Xie et al. 2011; Yildiz et al. 2013). These interventions presumed considerable control over minority members and were typically implemented by disguising confederates as minority members or inserting programmed computer agents in a group or network. A minority group composed entirely of entirely such individuals does not truly function as an organic union because they operate in isolation and do not exchange information with each other as normal group members do.

In contrast, we applied a rather subtle intervention by assembling isolated individuals with similar non‐mainstream opinions in a social network into a minority group. This approach increases the relational density among minority members, changes their sources of social influence, and makes them more likely to be influenced by fellow minority members than the majority. It is an intervention similar to *nudge*, techniques that employ non‐coercive methods to veer people's decisions toward certain directions (Thaler and Sunstein 2008). Our results show that this minority clustering intervention could indeed strengthen the solidarity of the minority members while simultaneously making their opinions more impactful on other members of the network. In general, it has three notable advantages over prior methods.

### 4.1 Indirectness

Minority clustering requires no explicit communication among individuals in a social network, adjusts only a small proportion of the network connections, and feeds individuals with genuine responses produced by real people. These should reduce the likelihood that those being intervened notice the intervention. According to the theory of reactance (Brehm 1966), when individuals perceive their freedom of choice is being restricted or controlled by external forces, they experience a *reactance* response aimed at resisting the loss of control and restoring their sense of autonomy. Minority clustering can circumvent reactance, allowing people to perceive their decisions as self‐directed rather than externally imposed.

### 4.2 Endogeneity

Minority clustering first identifies individuals who hold extreme yet similar opinions, then connects them to form a minority group. As such, the minority stance originates organically from within. It follows a natural developmental trajectory over time and thus has greater logical coherence, cultural fit, and experiential familiarity than externally imposed opinions. According to social identity theory (Hogg and Abrams 2007), people tend to favor in‐group members and sometimes display hostility toward out‐group members. Therefore, endogenous opinions typically invoke less resistance than exogenous ones. Moreover, different from introducing a wholly novel stance from scratch, an endogenous extreme opinion already enjoys local support within the group.

### 4.3 Spontaneity

With minority clustering, because individuals in a network do not cooperate knowingly—and often do not even realize they are part of an intervention—their participation appears self‐motivated. Once the initial intervention successfully unifies the minority members, they naturally develop a sense of commitment to their shared opinion and willingly preserve it. Thus, the clustering intervention does not need to incur continuous cost and effort to sustain its effect. In addition, rather than a top‐down leader‐follower dynamic, minority members can become active contributors, generating new arguments and rationales to support their stance. What they do, in a sense, is a form of *self‐intervention*, combining creativity with conviction in maintaining their non‐mainstream opinions.

Holders of minority opinions are chronically surrounded by disagreeing others. Repeated information exchange and interpersonal interactions often cause hesitation in isolated minority members and make them yield to majority influence. Our results show that minority cohesion generally decreases over time, but minority clustering can slow down the rate of this decline. This effect may occur for two reasons: (a) all things being equal, individuals are more likely to be influenced by like‐minded peers (e.g., Sunstein 2018), and seeing such neighbors can boost confidence in their own stance and make them more resolute; and (b) sustained opinion stability can generate positive feedback loops, creating opinion echo chambers and thus reinforcing minority cohesion (Cialdini and Goldstein 2004).

The ultimate goal of minority clustering is to increase minority members' influence on the majority. In a social network, mere self‐preservation of the minority is insufficient to shift group opinions; the minority must actively influence the majority. We found that minority clustering could effectively enhance minority influence, which was clearly related to its positive effect on minority cohesion. Only when minority opinions persist can they be perceived by majority members and subsequently influence them. Therefore, minority cohesion and minority influence can be viewed as two sides of the same coin. Indeed, previous research of minority influence also adopted this view, either explicitly (e.g., Moscovici et al. 1969) or implicitly (Jayles et al. 2017), in their designs of minority influence interventions.

Our results show that over repeated interactions, opinions of minority members and majority members tended to converge toward the middle and display a pattern of opinion *depolarization* in all experimental conditions (Figure 2). Through computer simulations, Botte et al. (2022) showed that clustering could lead to the formation of echo chambers and opinion polarization of a network. This suggests that echo chambers would be more likely to form and polarization more severe in the clustering condition of our study. However, this pattern did not show up in our results. A likely reason is that we only applied clustering to minority members, and the manipulation did not fully isolate minority members from outside influence. As a result, although minority members exhibited greater cohesion than majority members, the segregation between the two groups was insufficient to create pervasive echo chambers and opinion polarization in the network.

With the clustering manipulation, the minority functions as a cohesive group that possesses an existence beyond individual members. Even if members are replaced one by one, the minority could carry on like a “ship of Theseus” (Plutarch 2001), continuously resisting majority influence as a joined force. In other words, overt and forceful manipulations in the past aimed to transform ordinary people into extraordinary ones that are impervious to external influence (“supermen”), whereas the clustering manipulation assembles ordinary people into a solidary collective that spontaneously maintains minority opinions (“supergroup”). The power held by the latter lies in its numbers and organic growth.

Another intervention we examined is filter bubbles, corresponding to the common practice by most of the social media platforms (e.g., Flaxman et al. 2016). We found that filter bubbles could affect both minority cohesion and minority influence after more exchanges among network members. However, their effects were in opposite directions: It strengthened minority cohesion but diminished minority influence. These occurred because participants subject to filter bubbles manipulation were more likely to encounter neighbors whose opinions were similar to their own, and their positions were repeatedly reinforced as a result. Such reinforcements could help unify opinions and improve internal cohesion for both minority and majority members, making all members less susceptible to external influences. Continuing for some time without further intervention, filter bubbles would likely lead to scattered but closed small groups across a network, each holding their own particular views on a topic.

### 4.4 Contributions and Limitations

Our study shows that minority clustering could effectively enhance internal cohesion and potential influence of the minority members. Additionally, our results highlight the dual role of the filter bubble, demonstrating both its facilitative effect on minority cohesion and inhibitory effect on minority influence with repeated network interactions. Our use of simulated social networks demonstrates a more ecological and relevant way through which researchers could study and understand minority influence in the formation of public opinions, and our findings can provide valuable insights to platform designers and policymakers on how to disseminate minority opinions effectively in the digital age.

That said, our study and its results have several limitations. First, the number of questions examined and the number of interaction rounds in our experiment were relatively small, which may constrain the reliability of our results and prevent the investigation of long‐term opinion dynamics. Second, in many aspects, the networks in our study are much simpler than real‐world social networks, potentially limiting the external validity of our findings. Third, the questions asked in our study were relatively narrow in scope, raising the question of whether similar effects would generalize to more emotionally charged or socially contentious issues, such as moral dilemmas or health‐related controversies. Lastly, although the clustering manipulation did enhance minority cohesion and influence, it did not lead to a reversal of public opinion. This suggests that stronger or more sustained interventions are needed to allow minority members to alter group‐level outcomes more meaningfully.

### 4.5 Conclusion

The widespread use of social media has transformed people into nodes within social networks, where individuals continually influence each other and collectively shape the evolution and formation of group opinions (Cialdini and Goldstein 2004). In this study, we introduced minority clustering as a way to subtly and unobtrusively enhance minority cohesion and minority influence in a social network. Preserving minority voices and getting them heard is crucial for the diversity, productivity, and harmony of a group (e.g., Moscovici 1980; Page 2007). By capitalizing on spontaneous and natural group dynamics rather than artificially implanting stubborn individuals or agents, minority clustering provides a feasible and sustainable means to empower minorities and help build groups, organizations, and cultures that are stronger and more tolerant.

## Ethics Statement

This study was approved by the Ethics Committee of the Institute of Psychology, Chinese Academy of Sciences (approval number H20031). Written informed consent was obtained from each participant.

## Conflicts of Interest

The authors declare no conflicts of interest.

## Supporting information


**Data S1:** Supporting Information.

## Data Availability

The data that support the findings of this study are available from the corresponding author upon reasonable request.
