# Supplementary material for: Enhancing the Cohesion and Influence of Minority Opinions Through Clustering: A Social Network Experiment
Source: Psych J. 2025 Sep 21;14(6):940–51. doi: 10.1002/pchj.70051 (PMC12702592; doi:10.1002/pchj.70051)
Supplement: Supplementary file 1 — Data S1: Supporting Information. [file PCHJ-14-940-s001.docx]

**Supplementary Materials for**

**Enhancing the Cohesion and Influence of Minority Opinions through Clustering:**

**A Social Network Experiment**

**Part I. Number of Participants in Each Network Group**

There were four experimental conditions in our study, and four groups of participants in each condition. Table S1 reports the number of participants in each of the 16 groups.

**Table S1.** Number of Participants in Each Network Group

| Filter bubble | Clustering | Number of participants |
| --- | --- | --- |
| No | No | 27 |
| No | No | 26 |
| No | No | 30 |
| No | No | 29 |
| No | Yes | 28 |
| No | Yes | 26 |
| No | Yes | 32 |
| No | Yes | 25 |
| Yes | No | 29 |
| Yes | No | 26 |
| Yes | No | 29 |
| Yes | No | 32 |
| Yes | Yes | 29 |
| Yes | Yes | 28 |
| Yes | Yes | 29 |
| Yes | Yes | 31 |

**Part II. Distribution of Participant Opinions**

Each figure below shows the opinion distributions of a group of participants under a given experimental condition. In each figure, the title indicates the specific group (i.e., G1 to G4) in a certain experimental condition. In the title, Bubbles = 0 and 1 represent whether filter bubbles were absent or present, respectively; similarly, Clustering = 0 and 1 indicate the absence and the presence of the clustering manipulation, respectively. In each subplot, the *x*-axis is opinion value, and the *y*-axis the relative frequency.


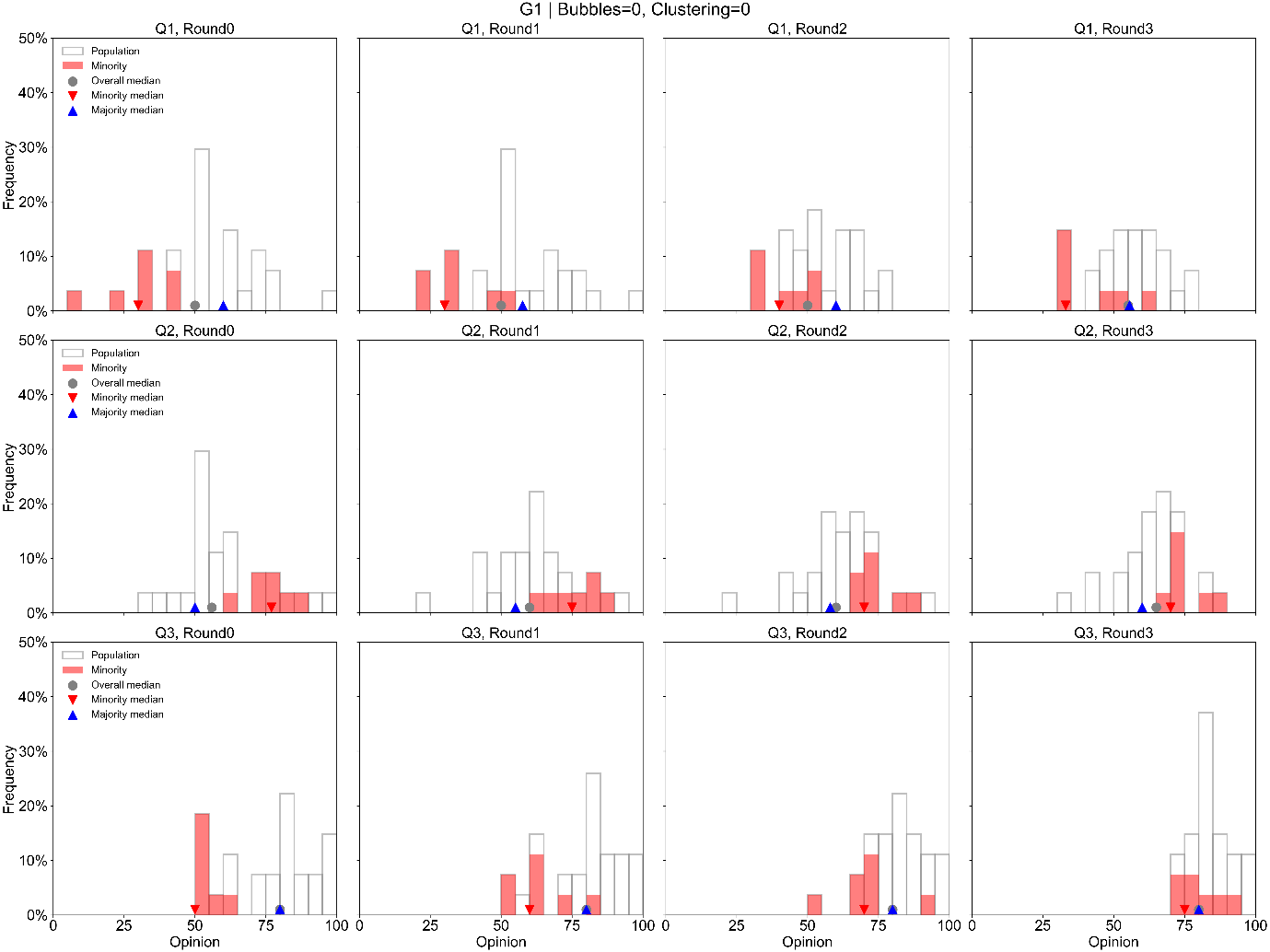


**Figure S1.** Opinion distribution of Group 1 participants under the condition without filter bubble and without clustering manipulation.


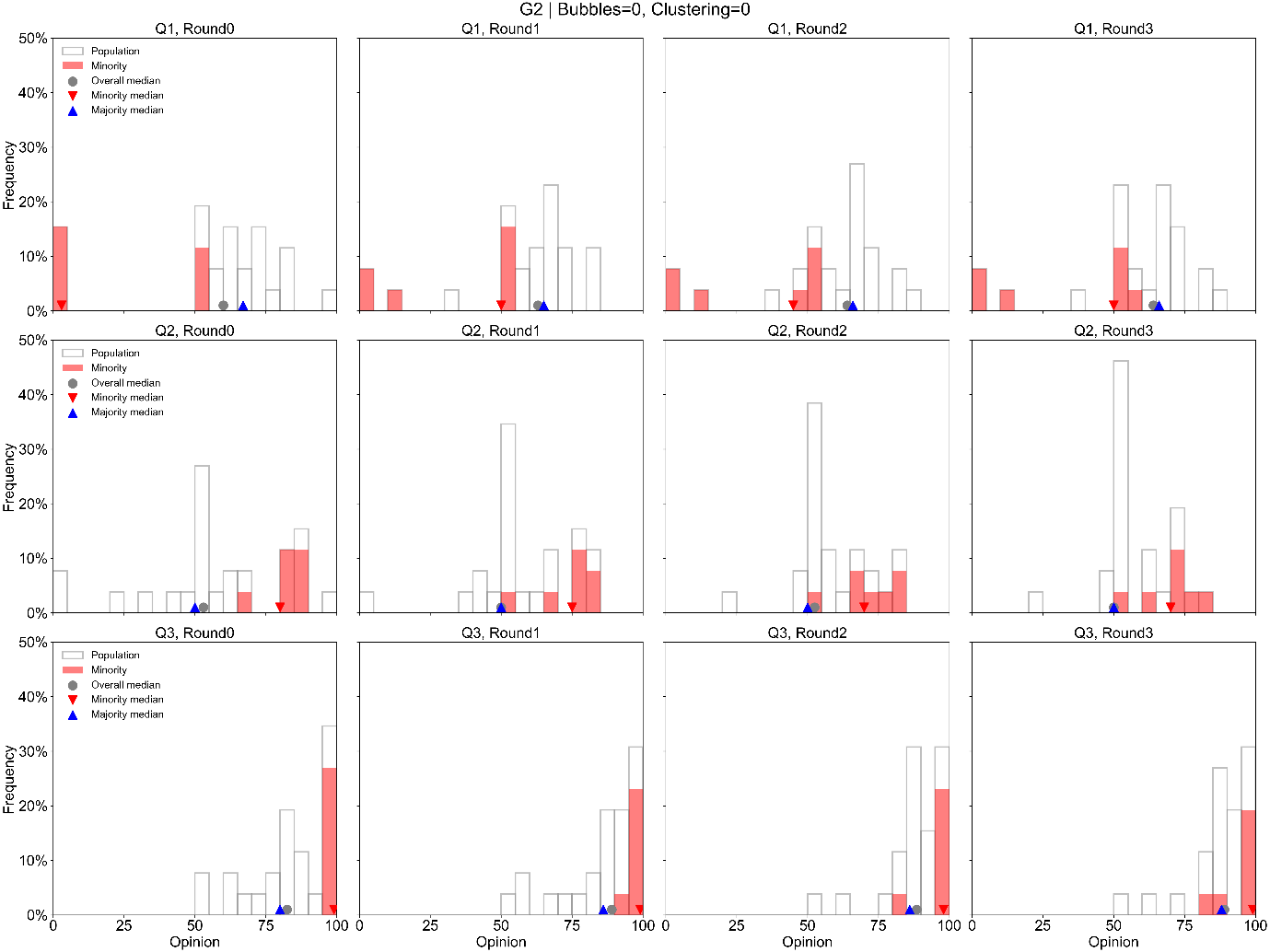


**Figure S2.** Opinion distribution of Group 2 participants under the condition without filter bubble and without clustering manipulation.


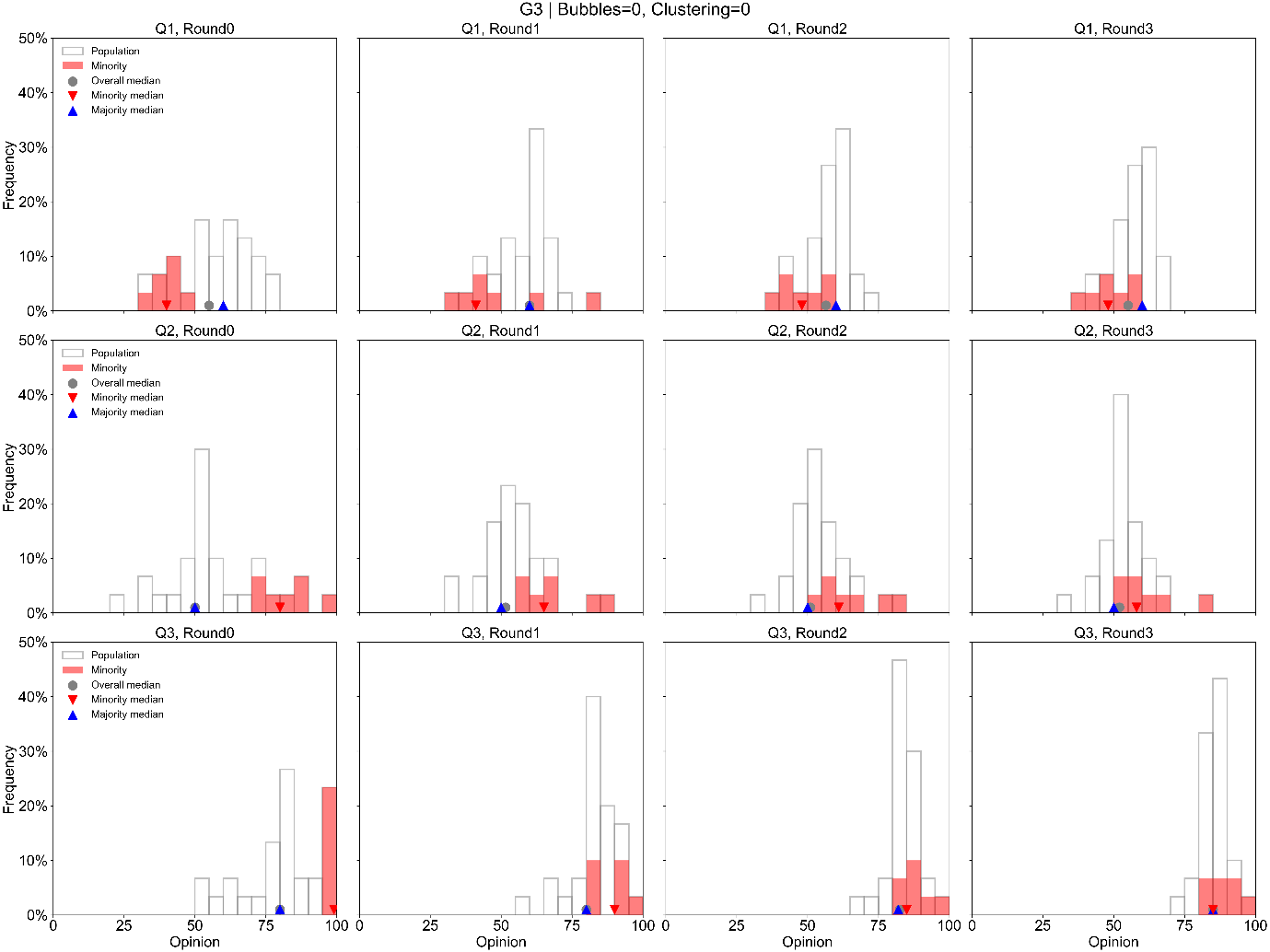


**Figure S3.** Opinion distribution of Group 3 participants under the condition without filter bubble and without clustering manipulation.


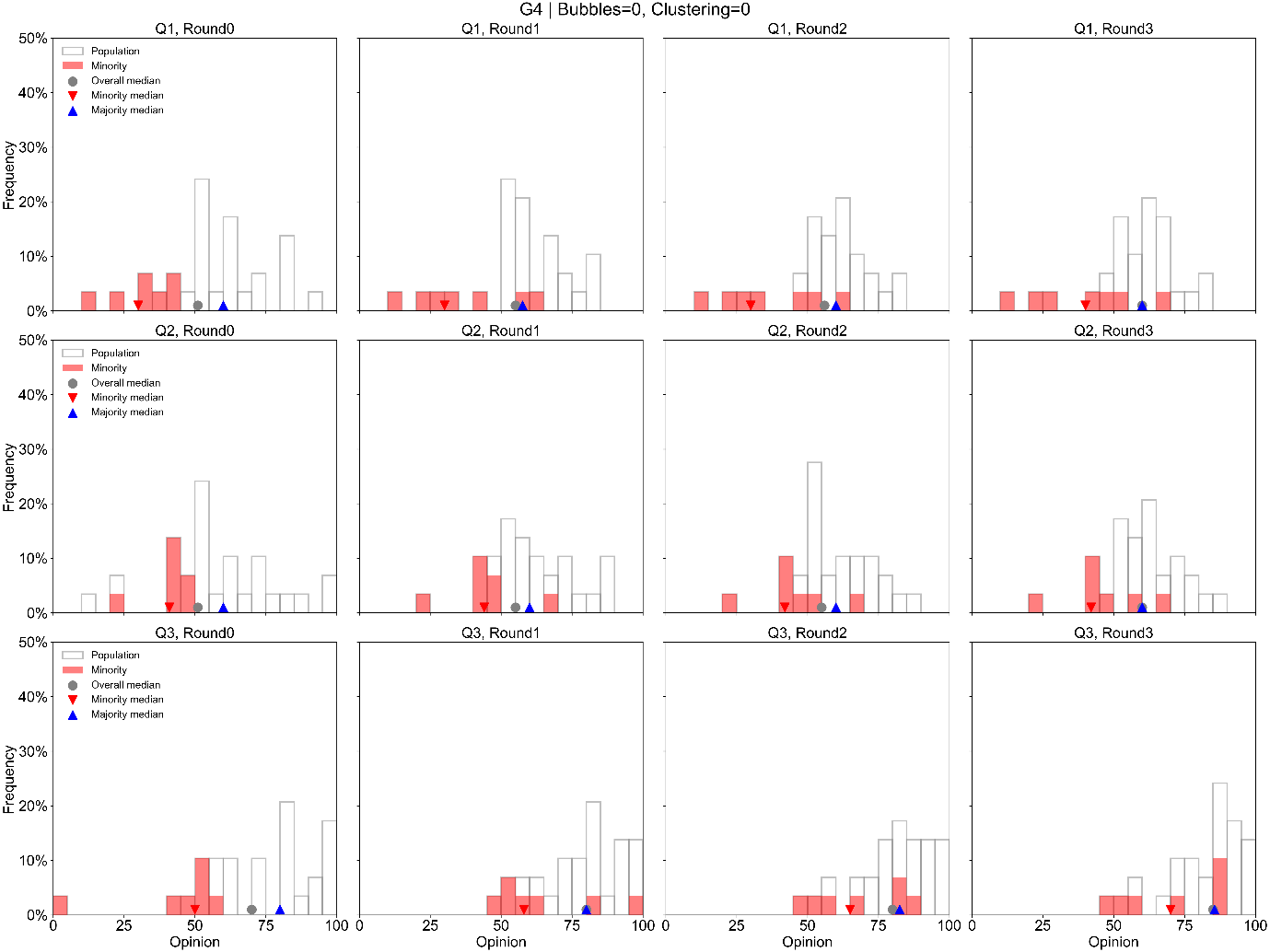


**Figure S4.** Opinion distribution of Group 4 participants under the condition without filter bubble and without clustering manipulation.


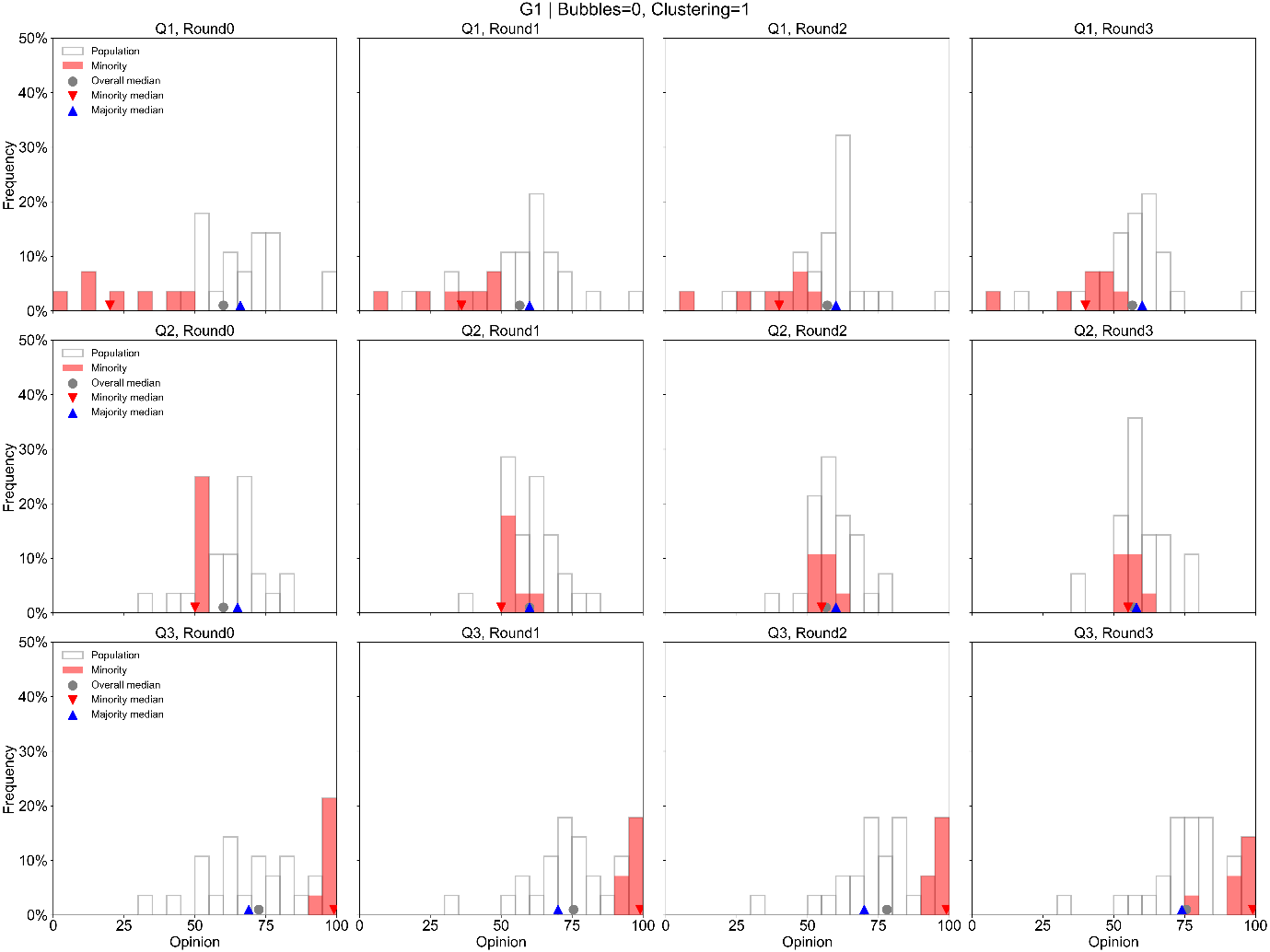


**Figure S5.** Opinion distribution of Group 1 participants under the condition without filter bubble but with clustering manipulation.


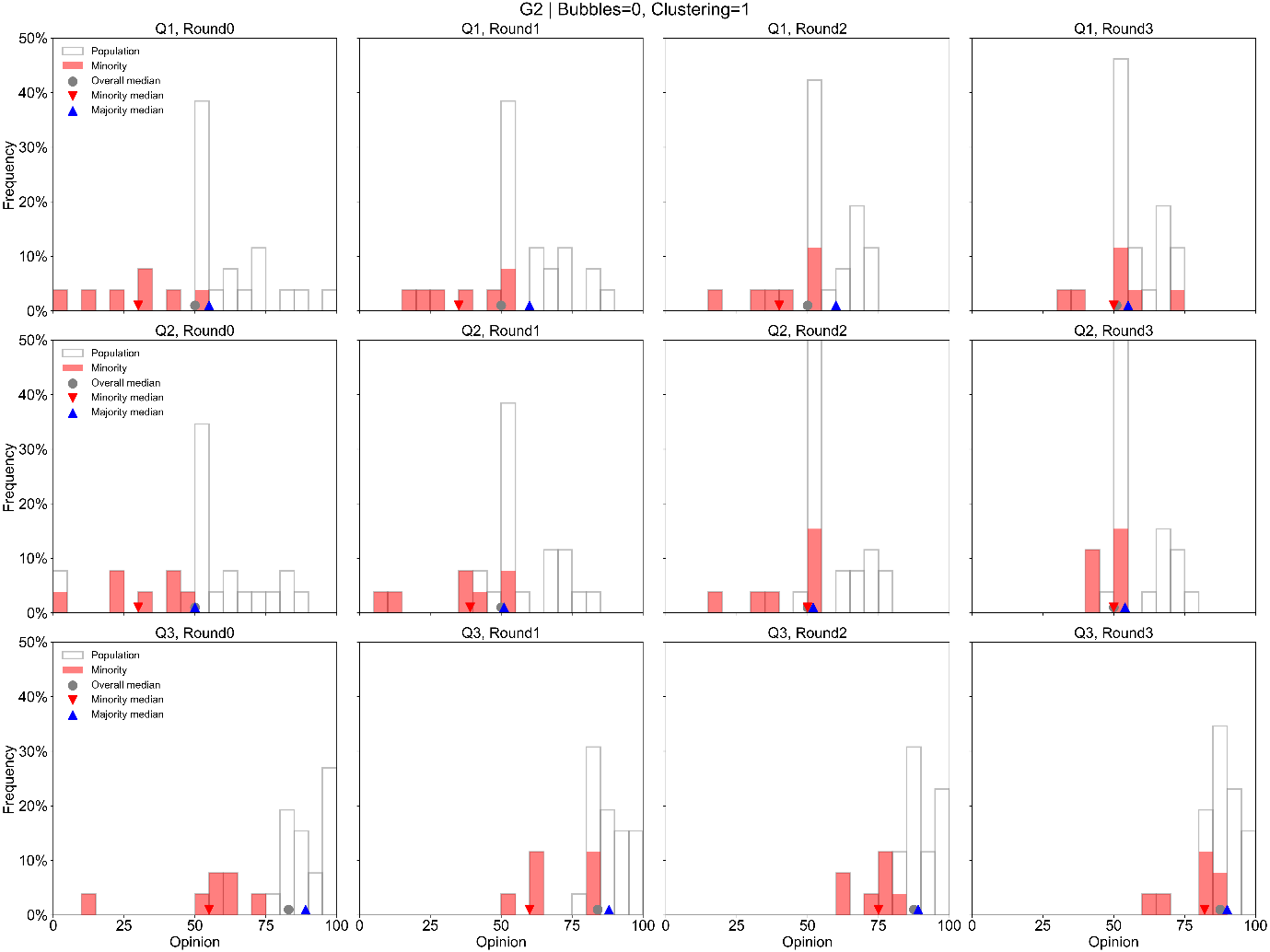


**Figure S6.** Opinion distribution of Group 2 participants under the condition without filter bubble but with clustering manipulation.


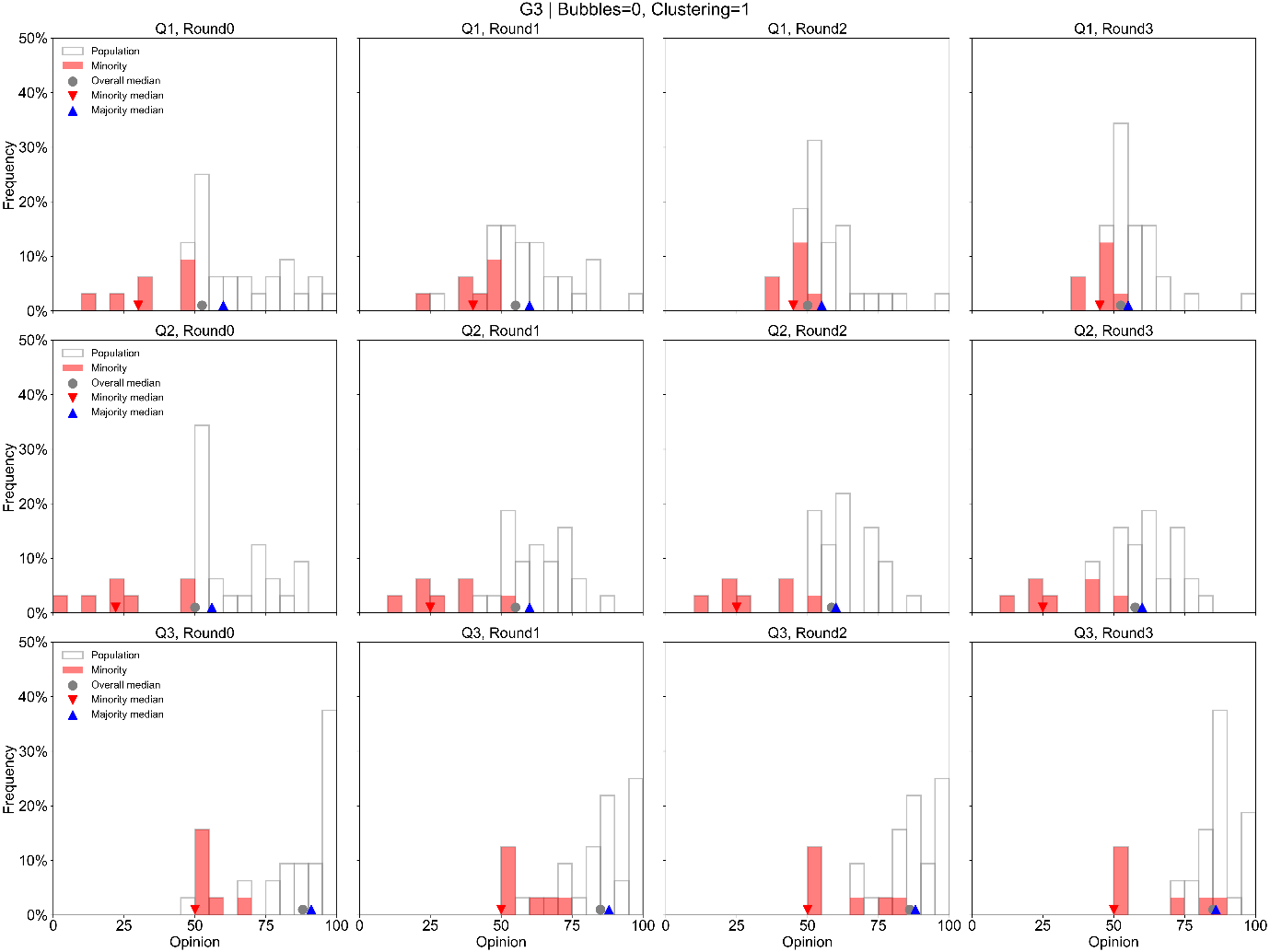


**Figure S7.** Opinion distribution of Group 3 participants under the condition without filter bubble but with clustering manipulation.


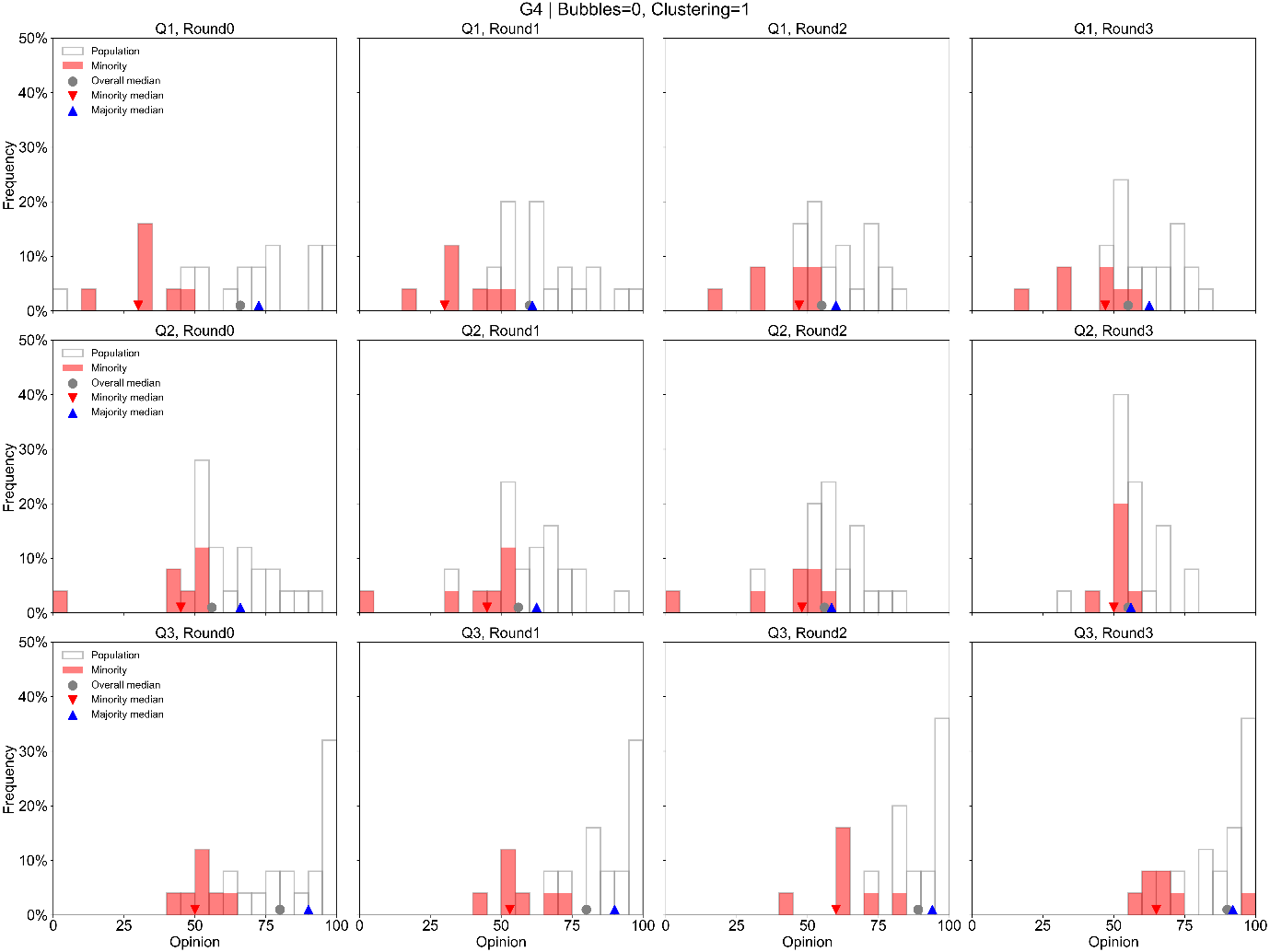


**Figure S8.** Opinion distribution of Group 4 participants under the condition without filter bubble but with clustering manipulation.


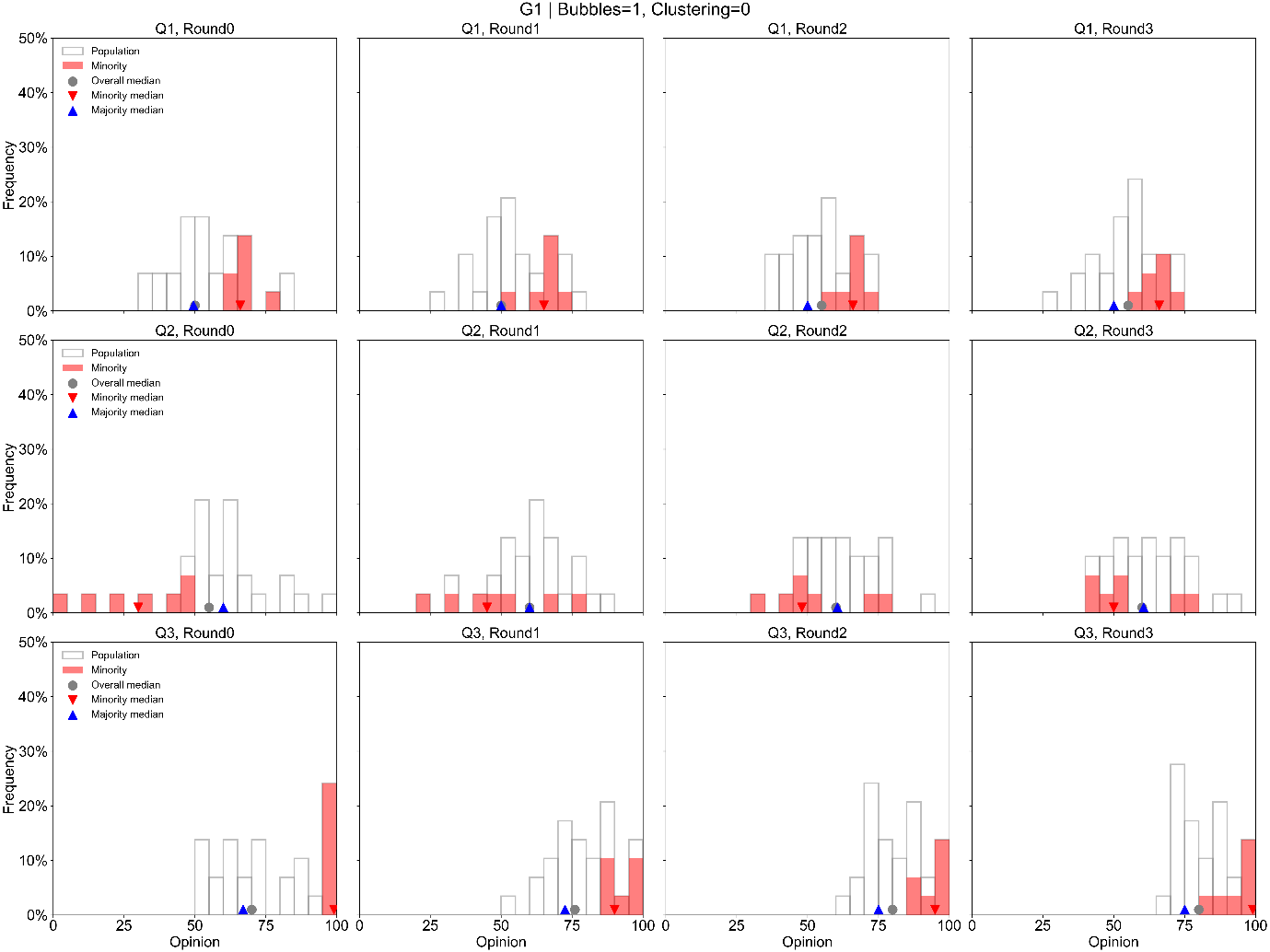


**Figure S9.** Opinion distribution of Group 1 participants under the condition with filter bubble but without clustering manipulation.


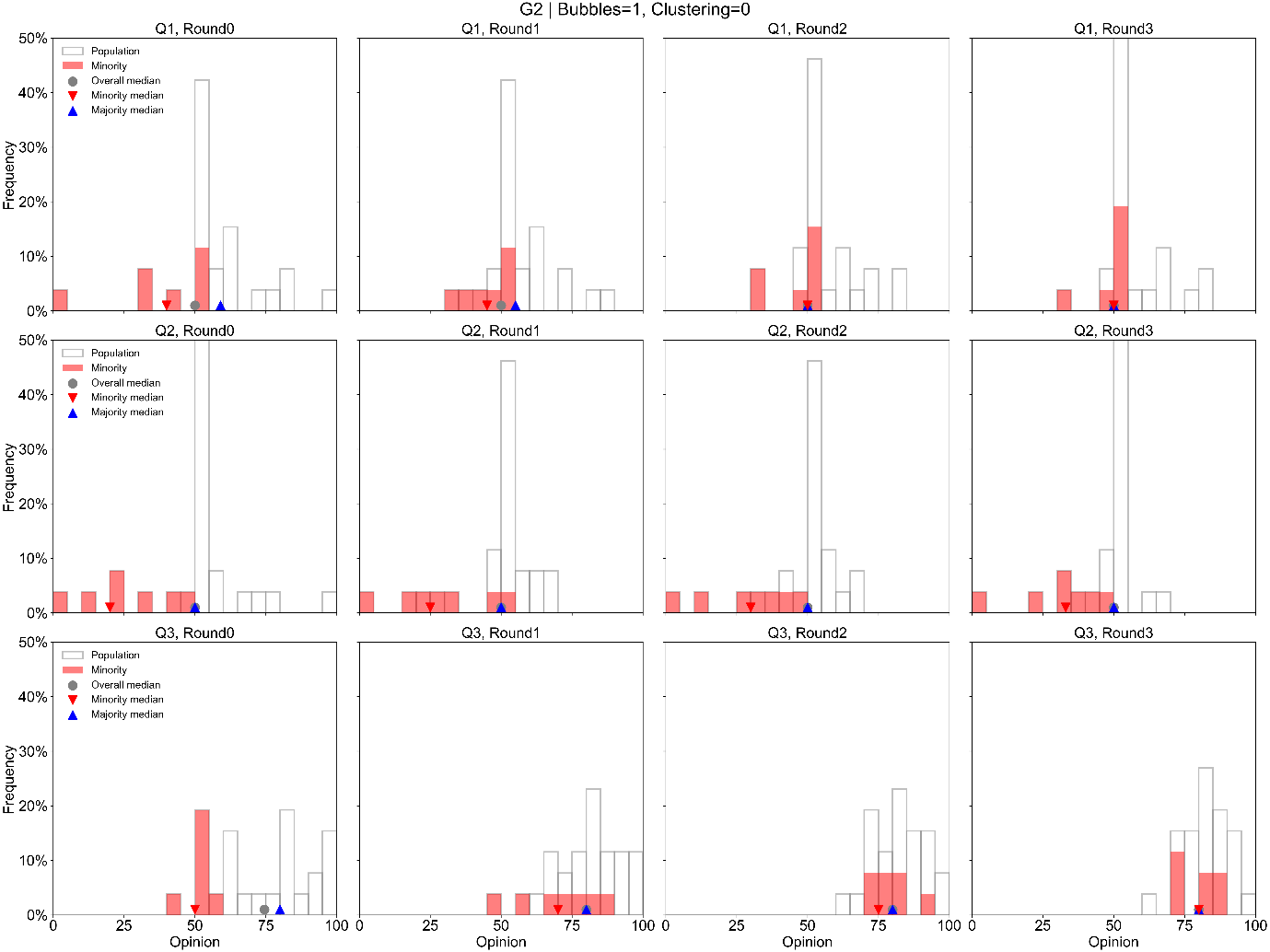


**Figure S10.** Opinion distribution of Group 2 participants under the condition with filter bubble but without clustering manipulation.


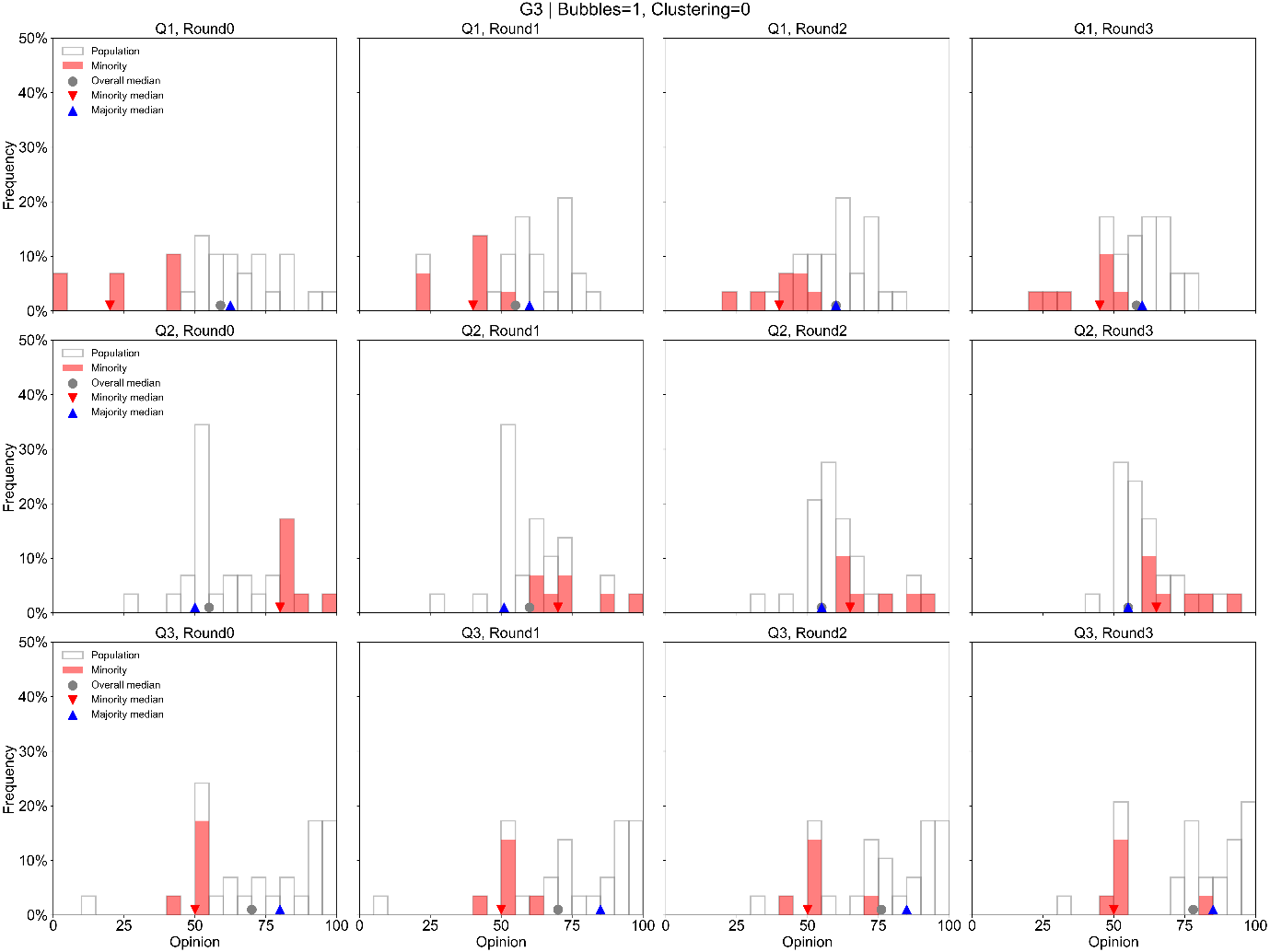


**Figure S11.** Opinion distribution of Group 3 participants under the condition with filter bubble but without clustering manipulation.

**
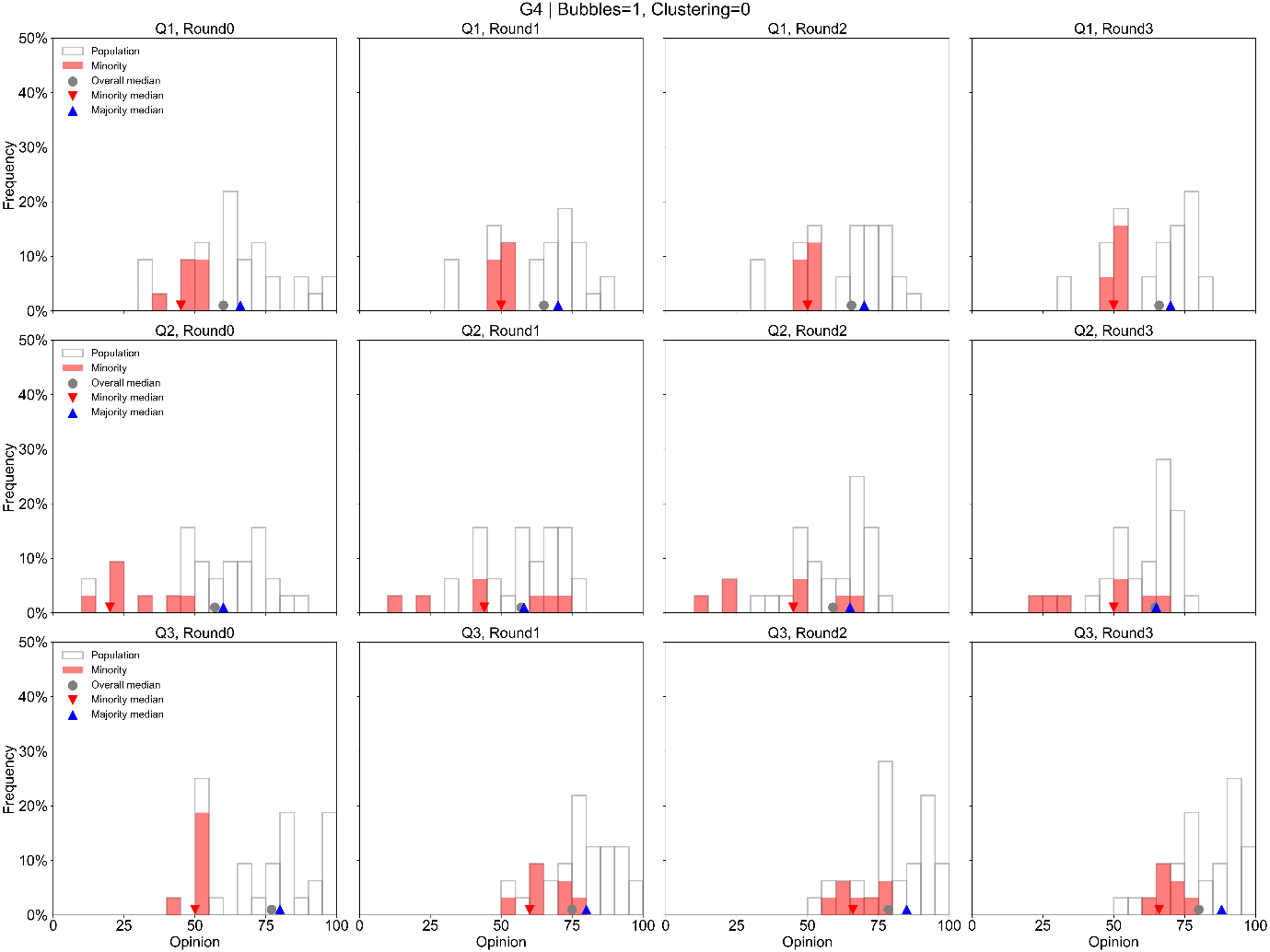
**

**Figure S12.** Opinion distribution of Group 4 participants under the condition with filter bubble but without clustering manipulation.

**
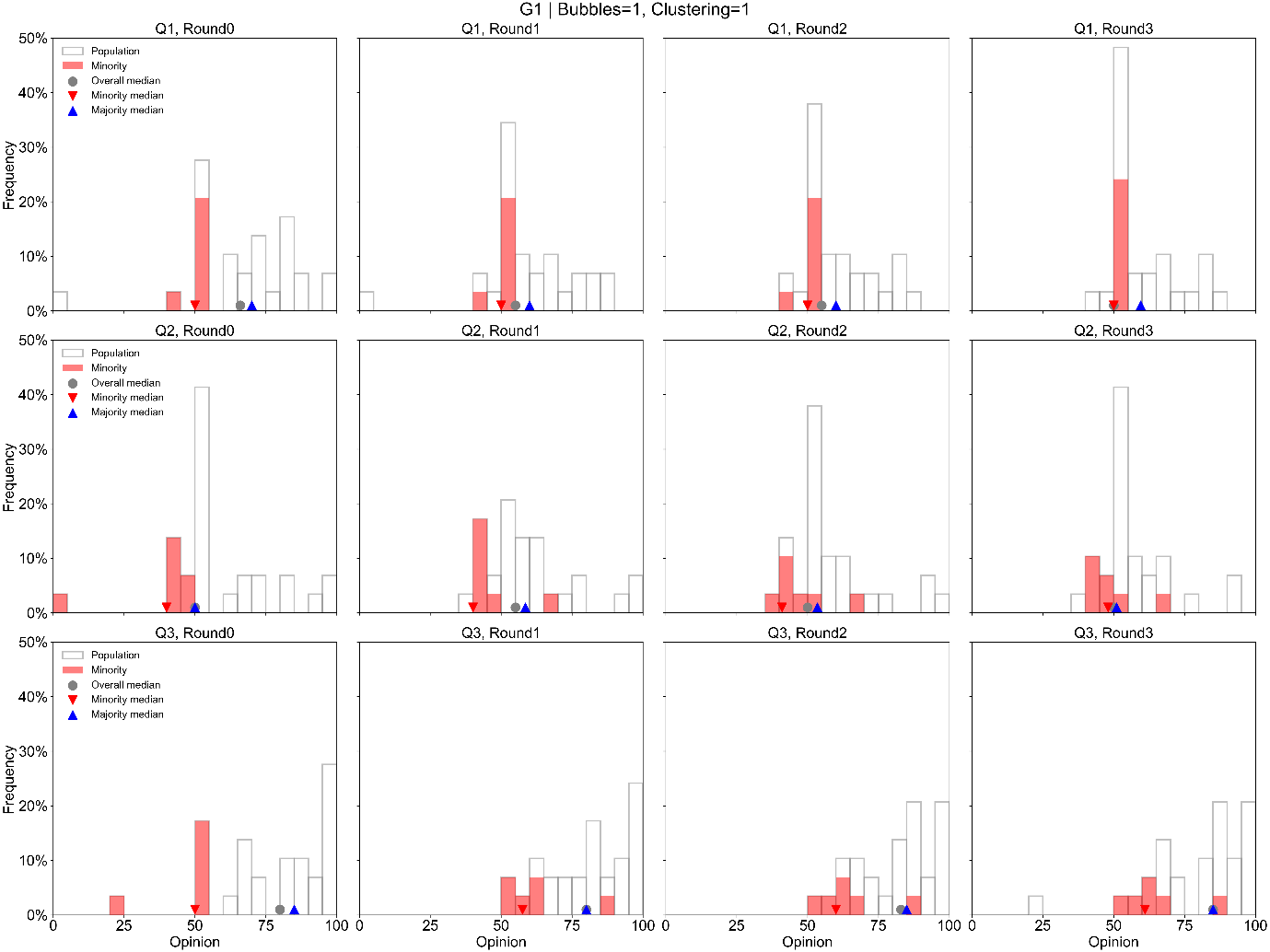
**

**Figure S13.** Opinion distribution of Group 1 participants under the condition with filter bubble and with clustering manipulation.

**
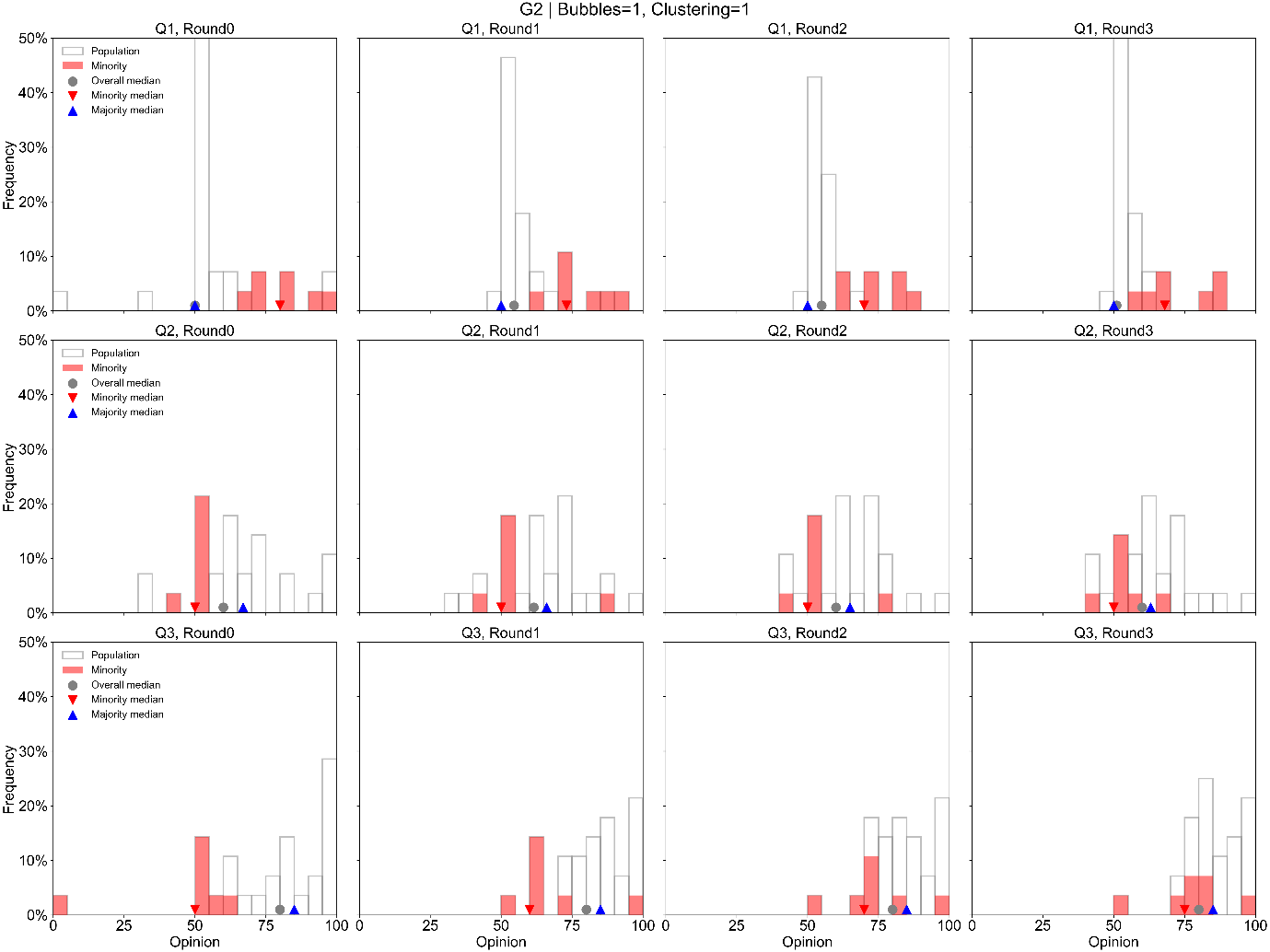
**

**Figure S14.** Opinion distribution of Group 2 participants under the condition with filter bubble and with clustering manipulation.

**
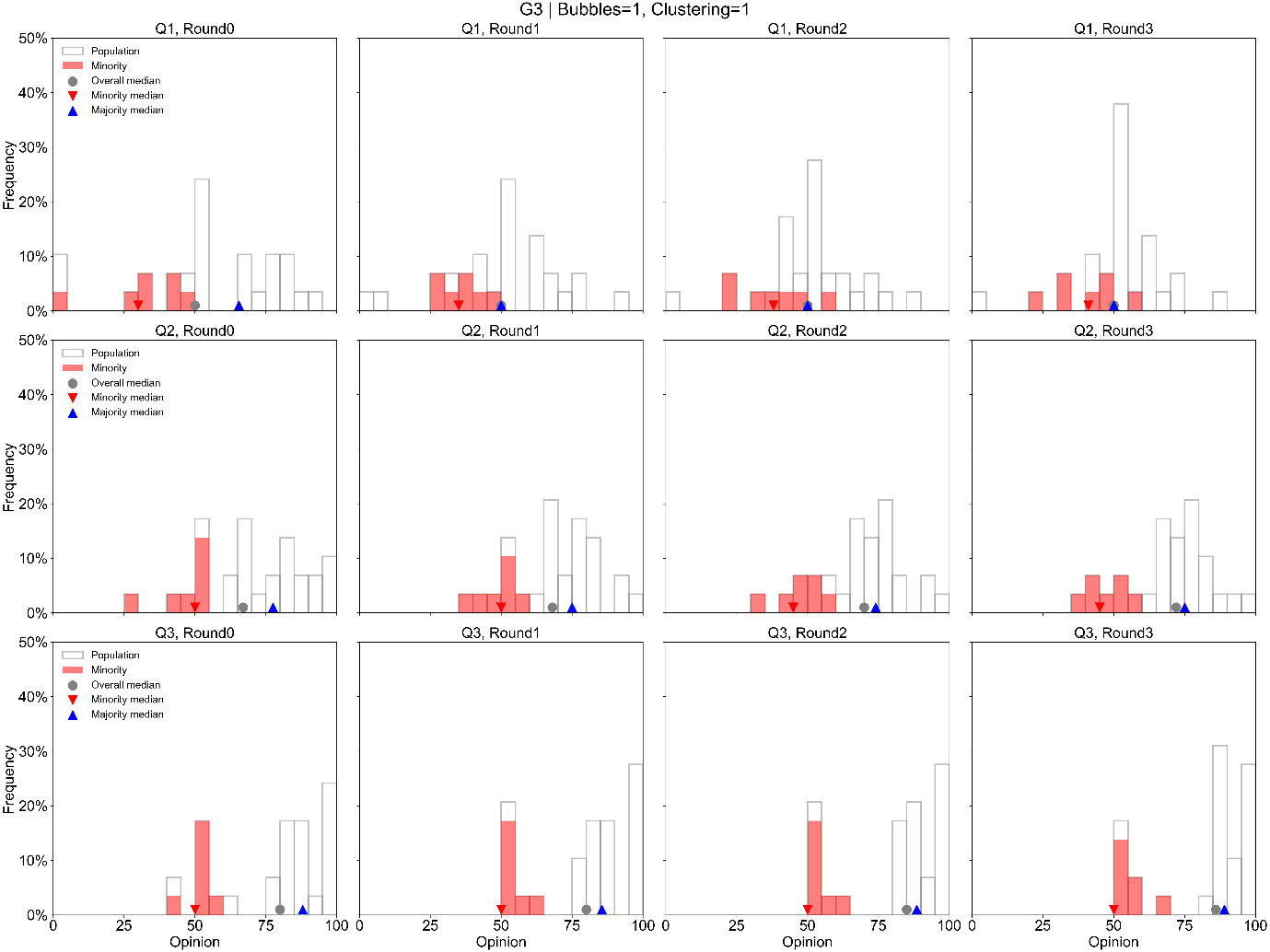
**

**Figure S15.** Opinion distribution of Group 3 participants under the condition with filter bubble and with clustering manipulation.


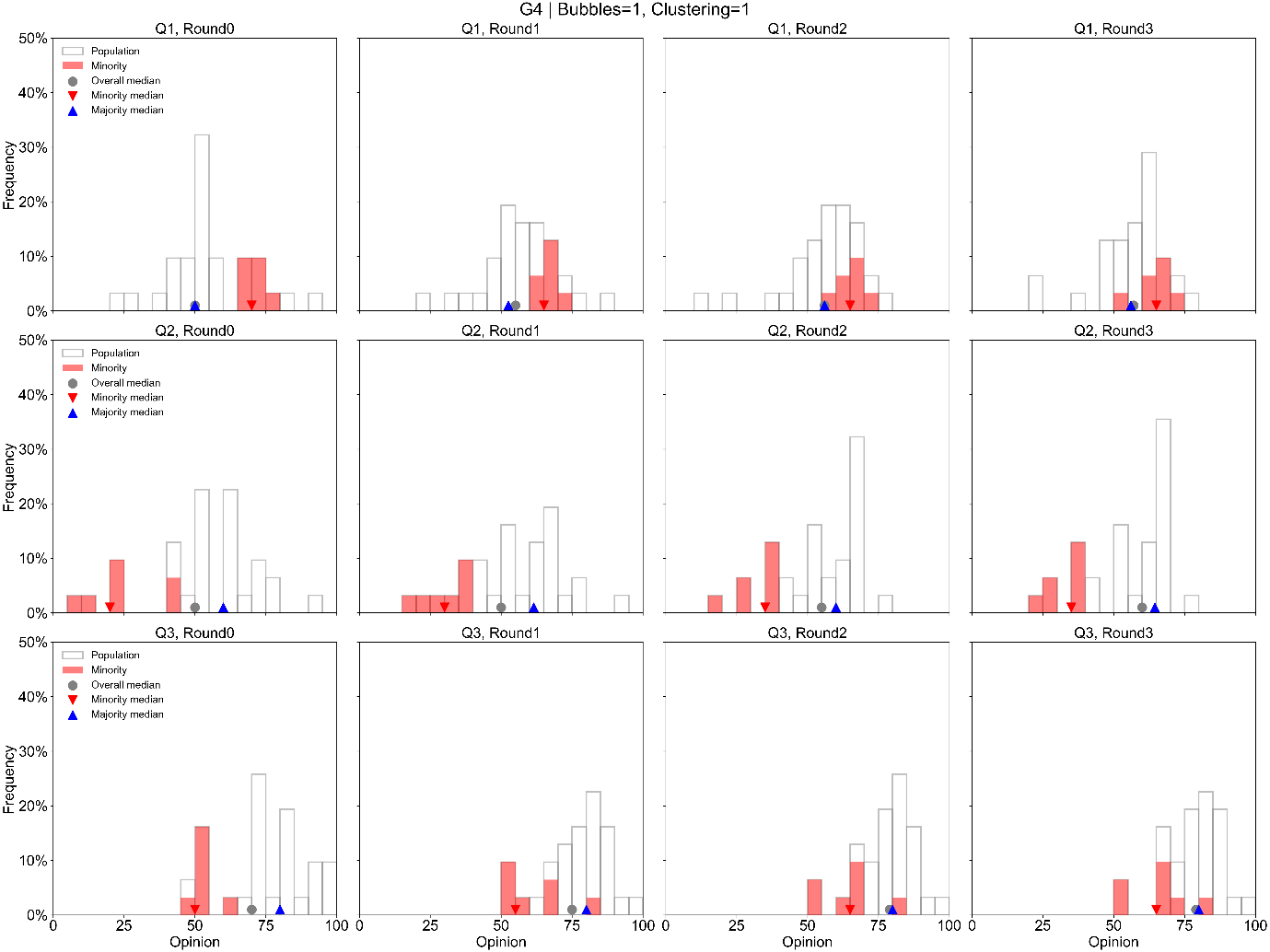


**Figure S16.** Opinion distribution of Group 4 participants under the condition with filter bubble and with clustering manipulation.

**Part III. Dynamic Opinion Evolution**

Each figure below shows the dynamic evolution of opinions for a group of participants in a given experimental condition. In each figure, the title shows the specific group (i.e., G1 to G4) and the experimental condition it was in. In the title, Bubbles = 0 and 1 represent whether filter bubbles were absent or present, respectively; similarly, Clustering = 0 and 1 indicate the absence and the presence of the clustering manipulation, respectively. In each subplot, the inner (and larger) nodes represent the seven minority members, and the outer (and smaller) nodes represent majority members. Arrows indicate the presence and directions of social influences in a round. The color of each node denotes the centralized opinion of a participant, which was calculated as the original opinion value minus 50 and thus gives a better illustration of opinion direction.


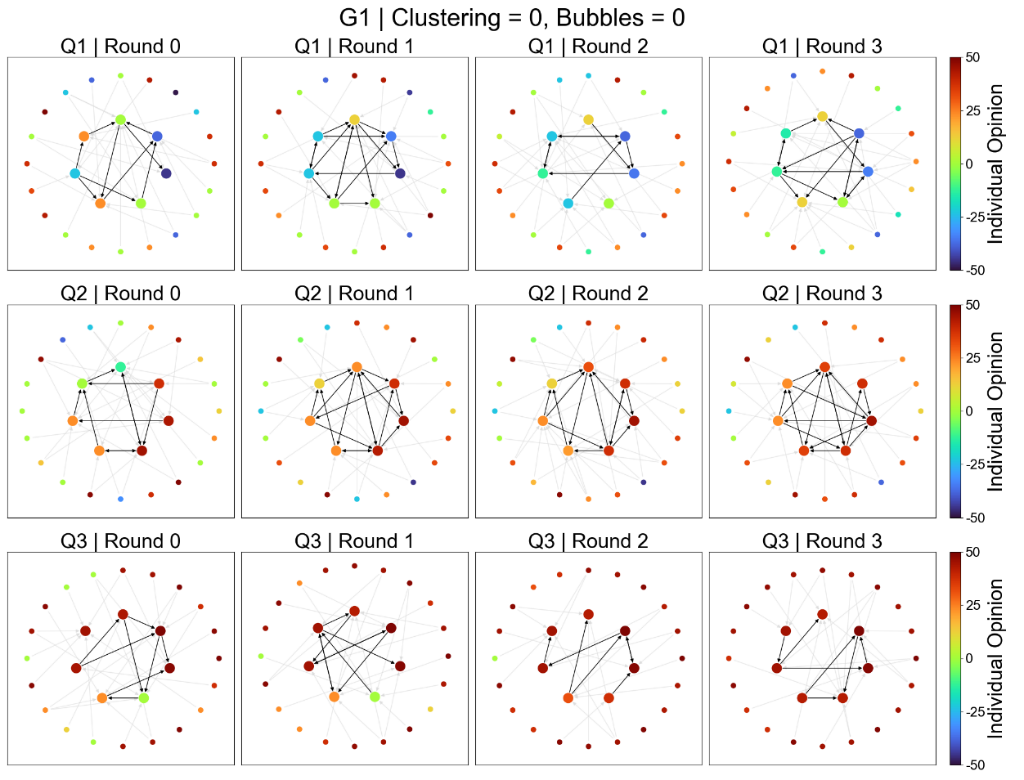


**Figure S17.** Opinion evolution of Group 1 participants under the condition without filter bubble and without clustering manipulation.


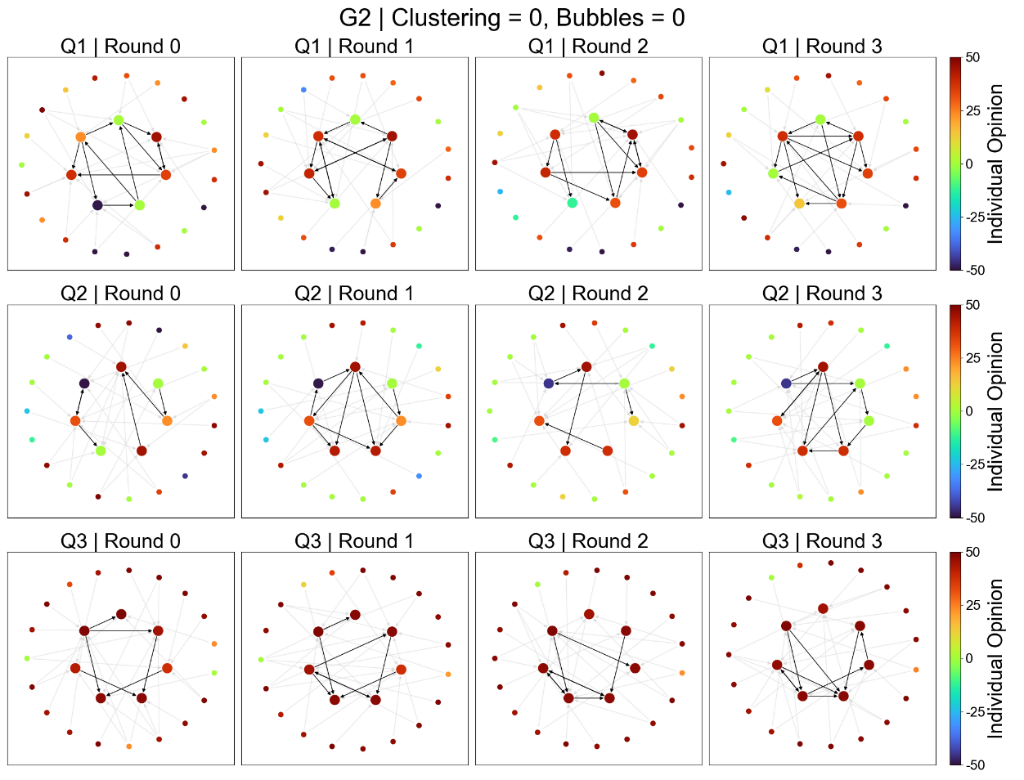


**Figure S18.** Opinion evolution of Group 2 participants under the condition without filter bubble and without clustering manipulation.


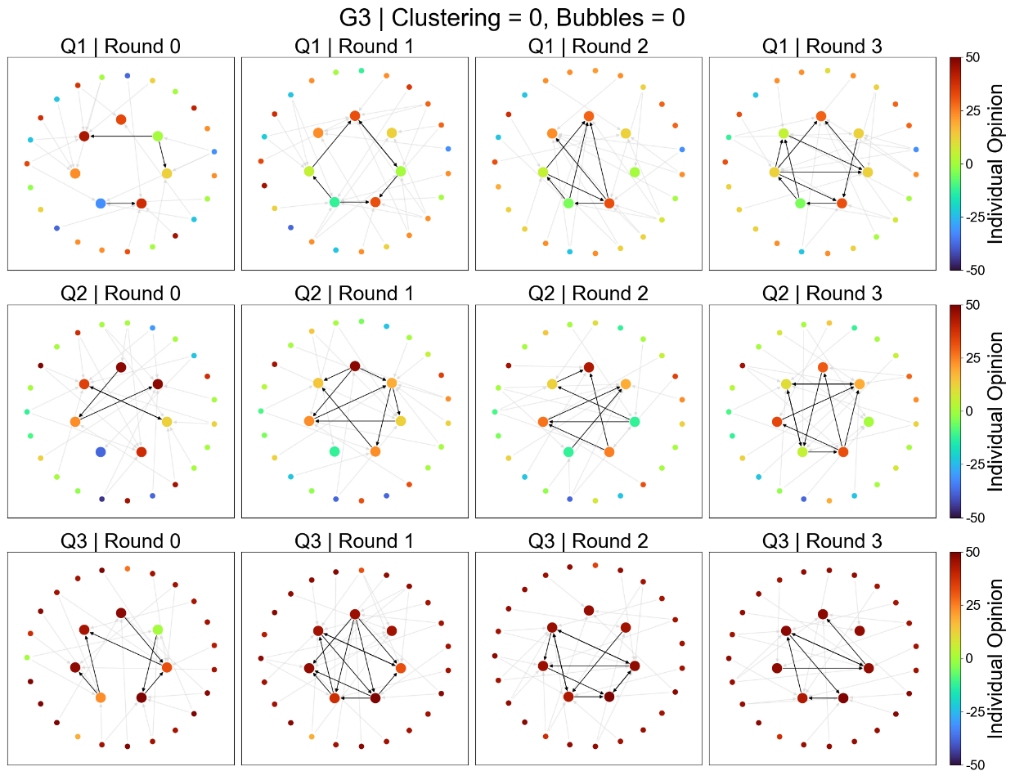


**Figure S19.** Opinion evolution of Group 3 participants under the condition without filter bubble and without clustering manipulation.


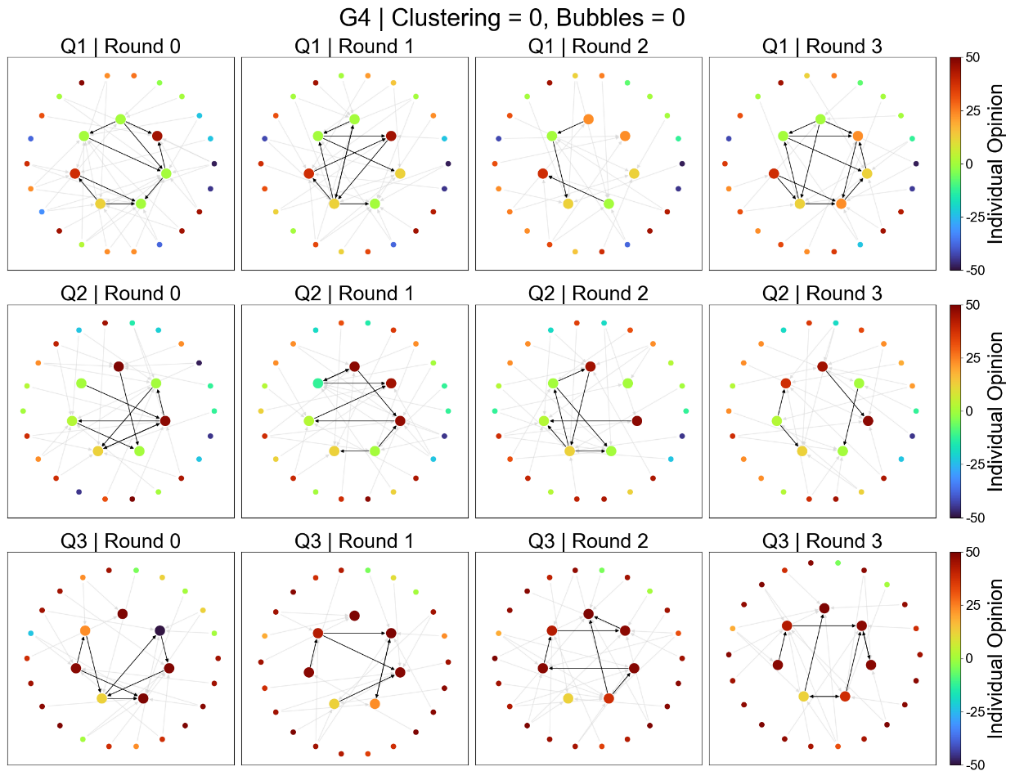


**Figure S20.** Opinion evolution of Group 4 participants under the condition without filter bubble and without clustering manipulation.


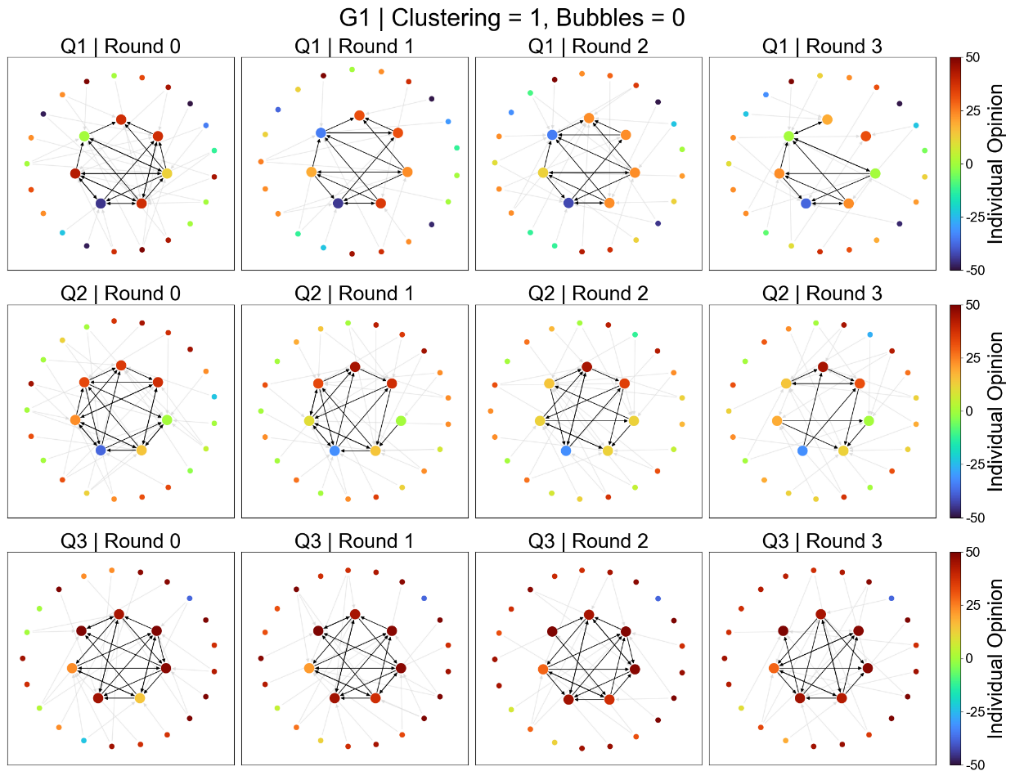


**Figure S21.** Opinion evolution of Group 1 participants under the condition without filter bubble but with clustering manipulation.


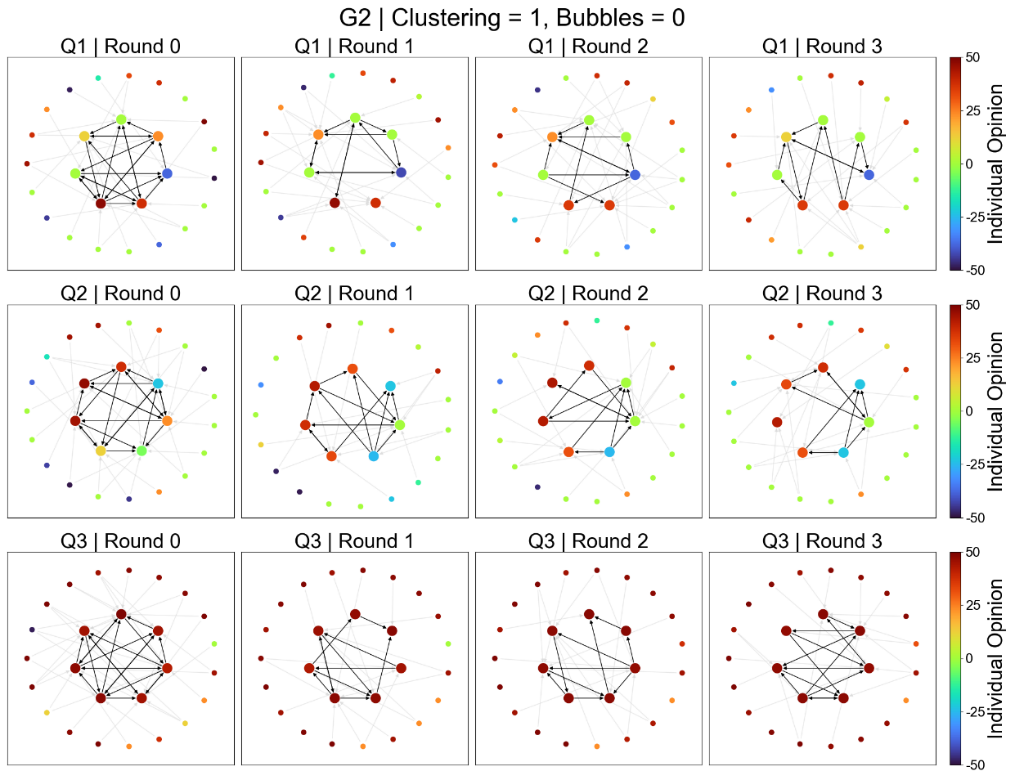


**Figure S22.** Opinion evolution of Group 2 participants under the condition without filter bubble but with clustering manipulation.


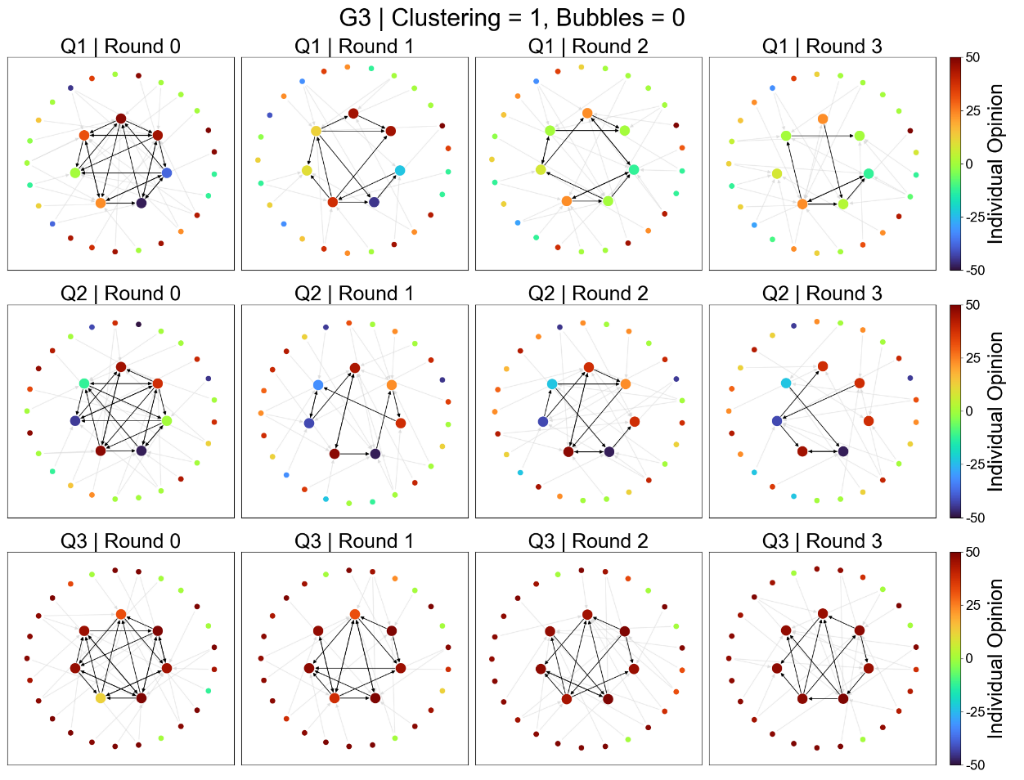


**Figure S23.** Opinion evolution of Group 3 participants under the condition without filter bubble but with clustering manipulation.


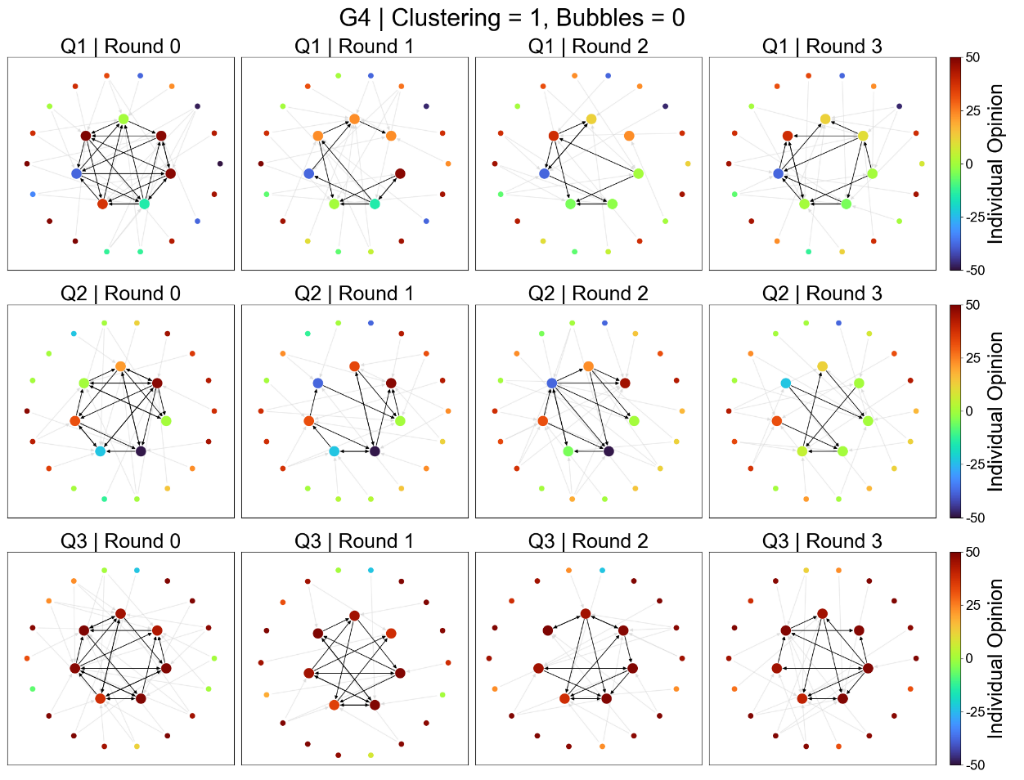


**Figure S24.** Opinion evolution of Group 4 participants under the condition without filter bubble but with clustering manipulation.


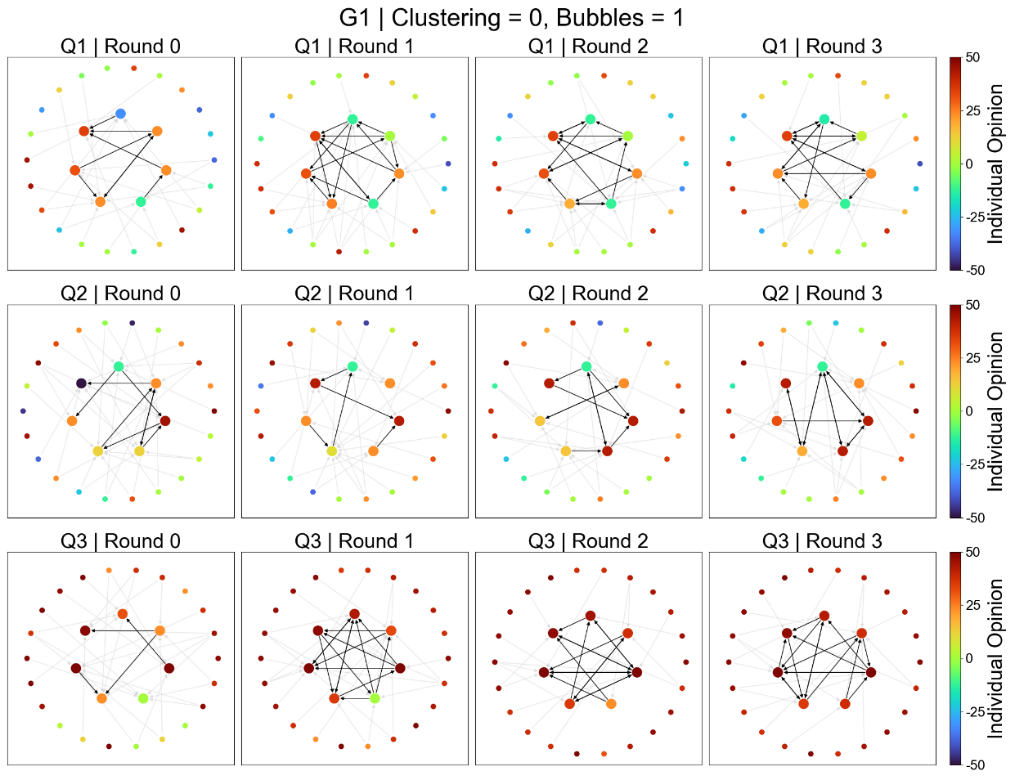


**Figure S25.** Opinion evolution of Group 1 participants under the condition with filter bubble but without clustering manipulation.


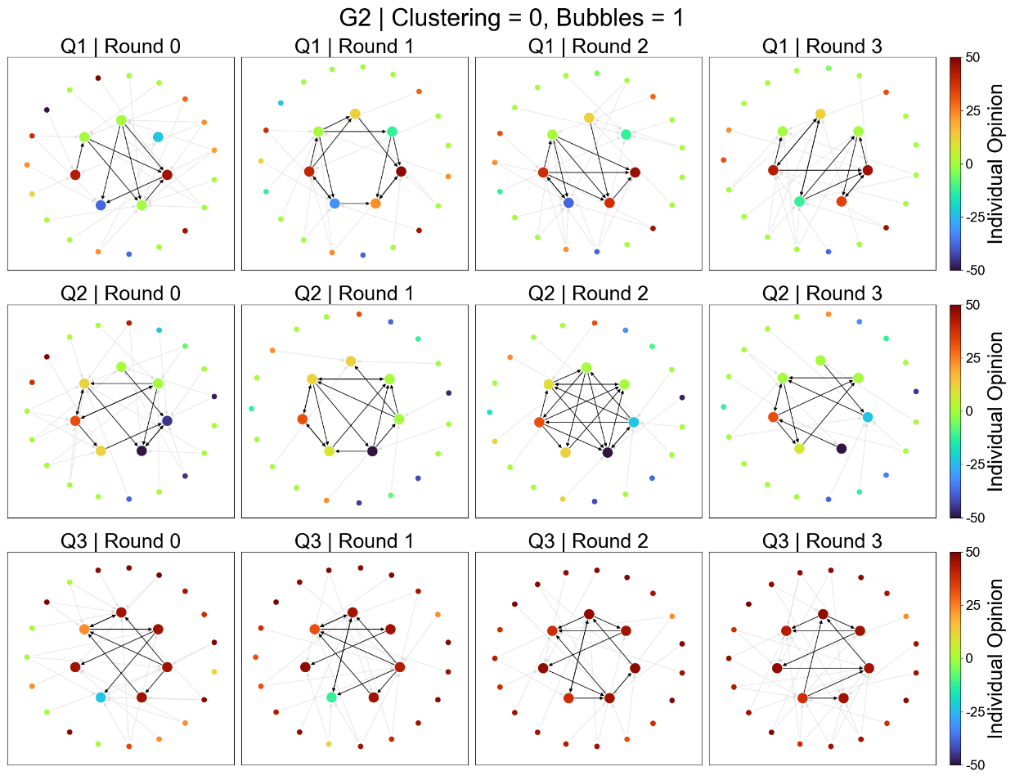


**Figure S26.** Opinion evolution of Group 2 participants under the condition with filter bubble but without clustering manipulation.


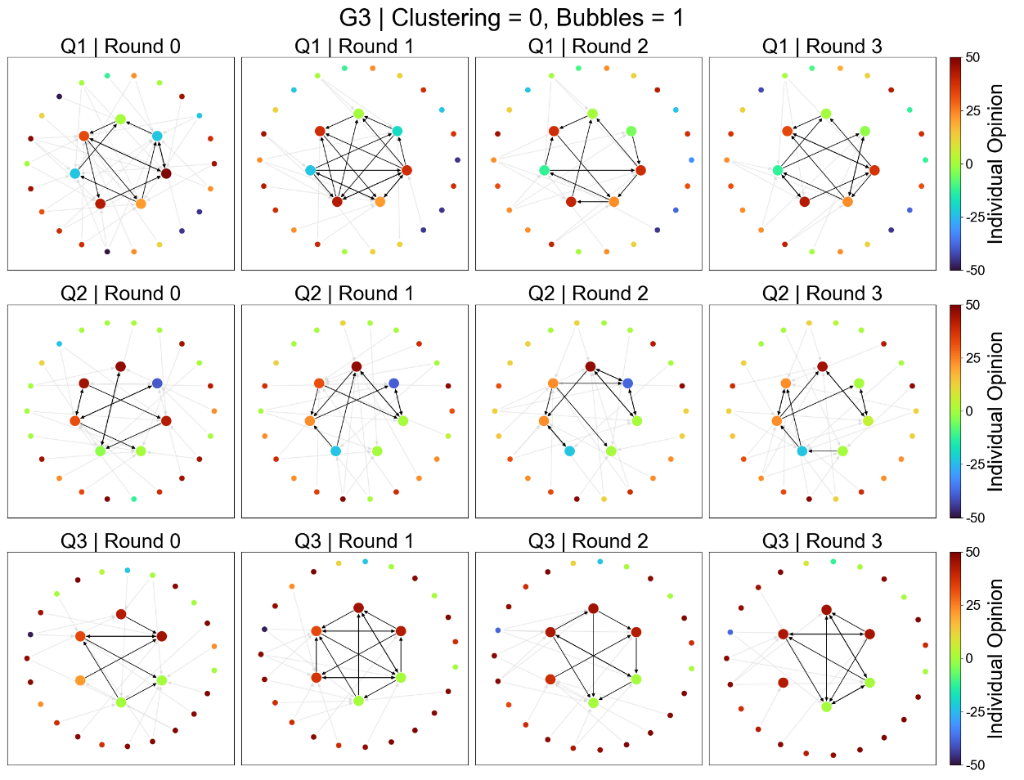


**Figure S27.** Opinion evolution of Group 3 participants under the condition with filter bubble but without clustering manipulation.


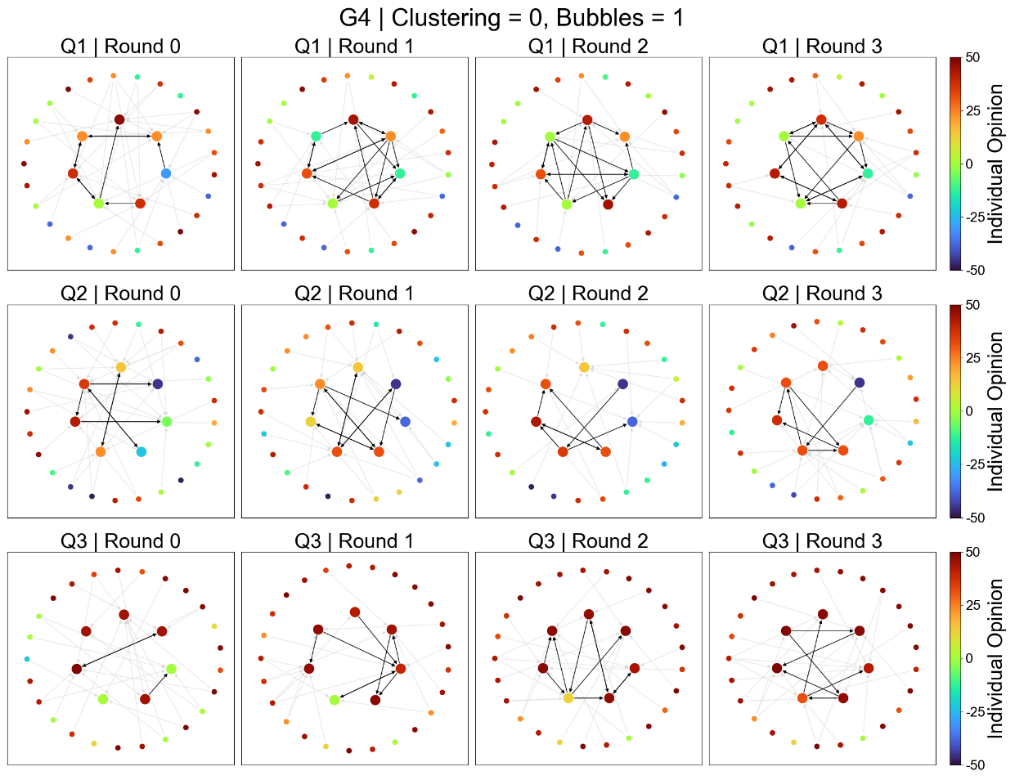


**Figure S28.** Opinion evolution of Group 4 participants under the condition with filter bubble but without clustering manipulation.


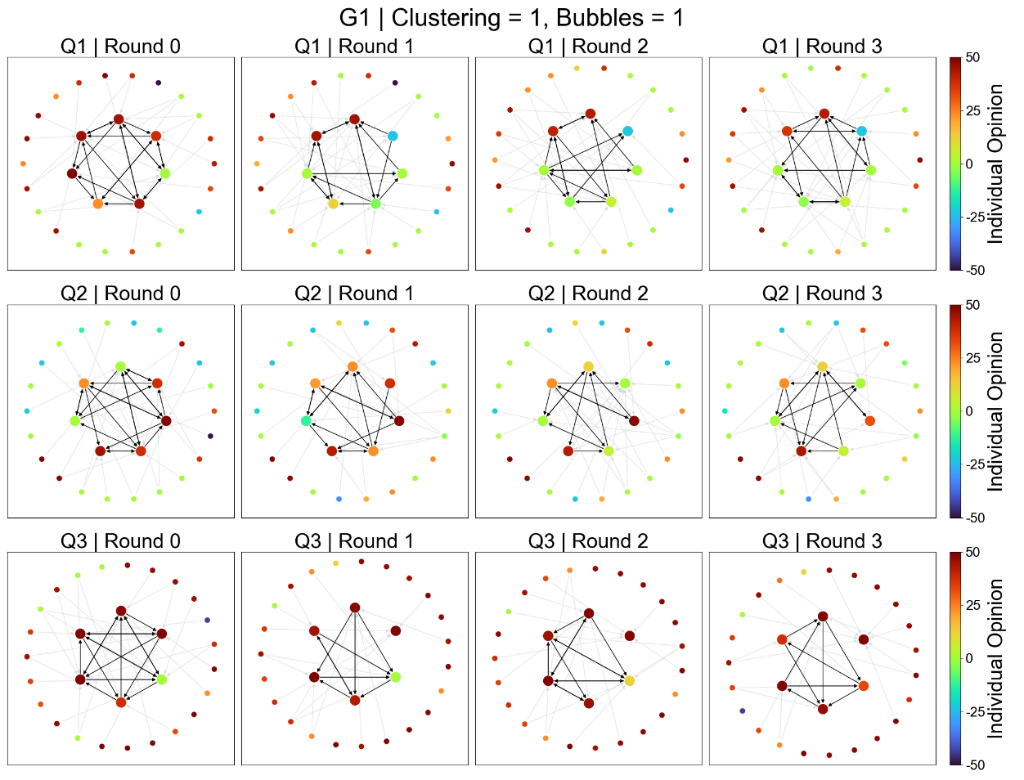


**Figure S29.** Opinion evolution of Group 1 participants under the condition with filter bubble and with clustering manipulation.


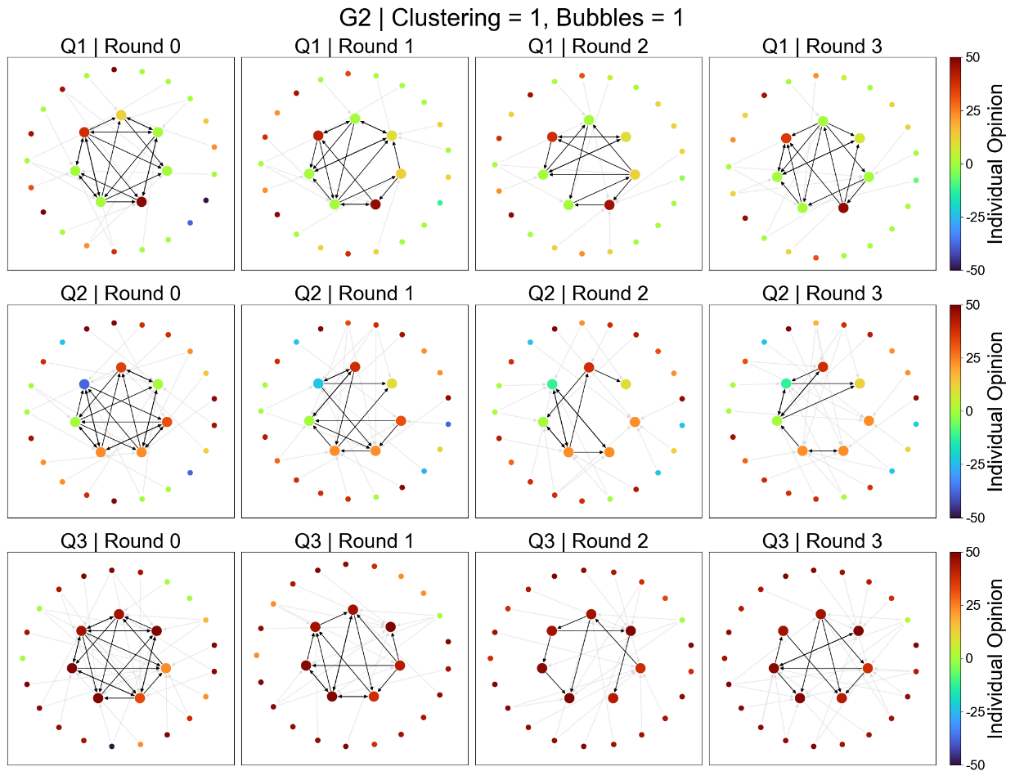


**Figure S30.** Opinion evolution of Group 2 participants under the condition with filter bubble and with clustering manipulation.


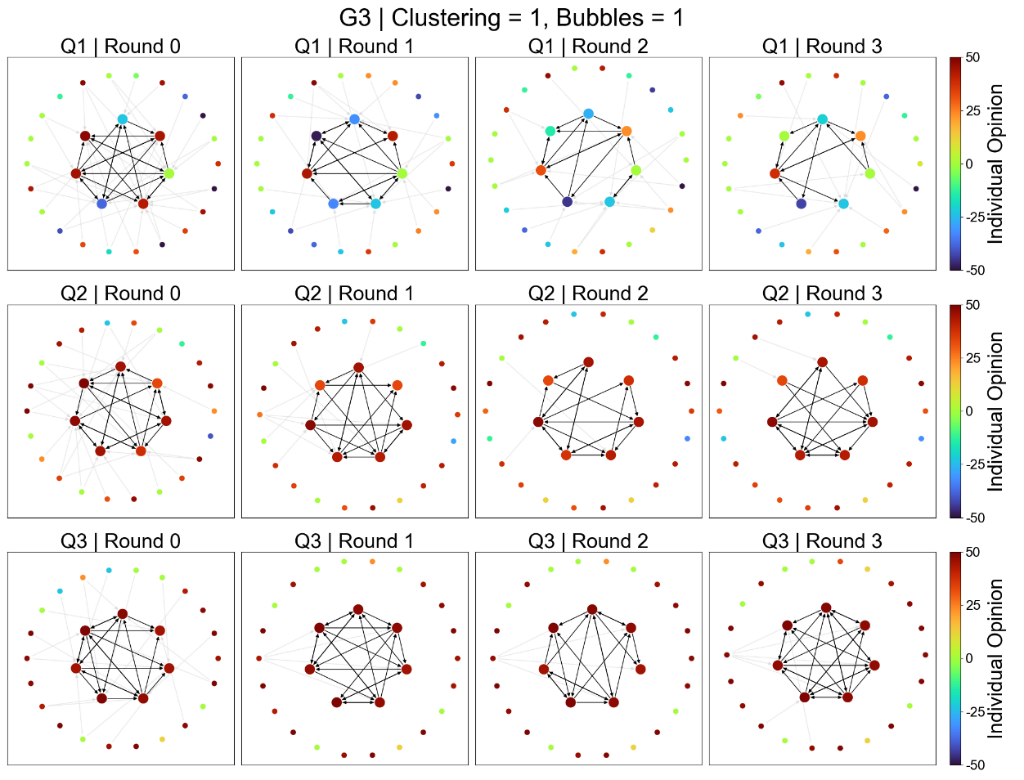


**Figure S31.** Opinion evolution of Group 3 participants under the condition with filter bubble and with clustering manipulation.


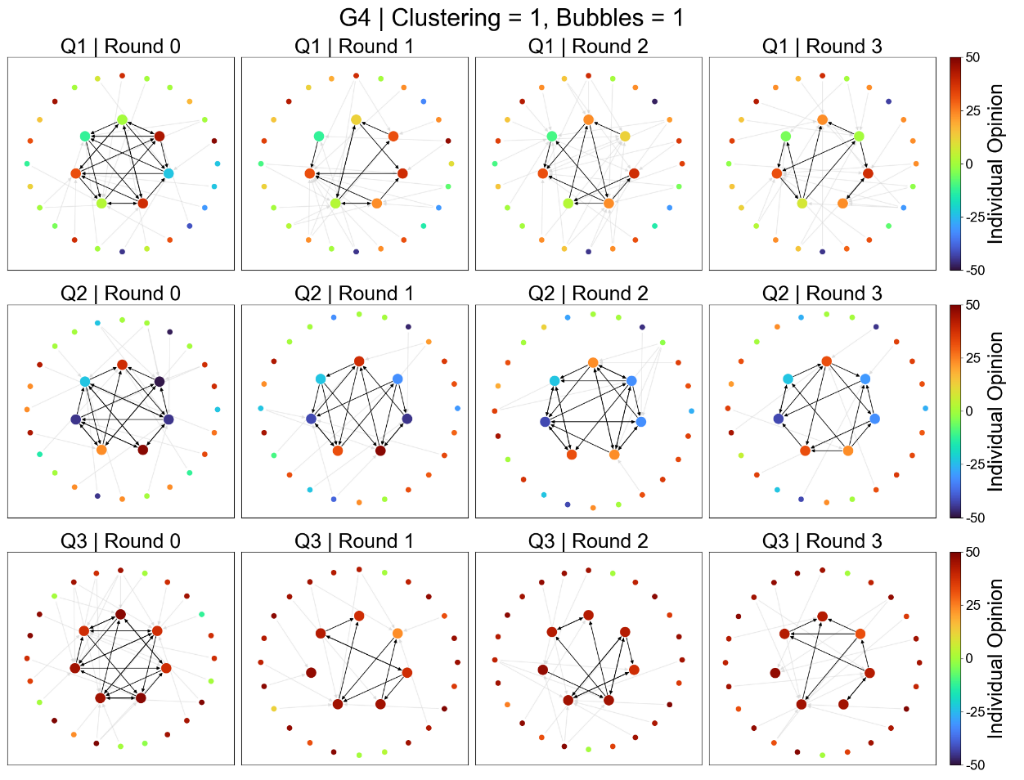


**Figure S32.** Opinion evolution of Group 4 participants under the condition with filter bubble and with clustering manipulation.

**Part IV. Model Fits of Mixed-Effects Models**

We ran a series of mixed-effects models that included the main effects of clustering, filter bubble, and round but with different combinations of interaction effects among the three variables. AIC and BIC were applied to judge the goodness of fit of each model. As shown in Table S2, for both the minority and the majority subgroups, the model including only the interaction between filter bubble (i.e., Bubble) and round yielded the lowest AIC and BIC values. The table also shows model fits of other models for the sake of comparison.

**Table S2.** Fits of Different Mixed-Effect Models

| Subgroup | Model | AIC | BIC |
| --- | --- | --- | --- |
| Minority | Three two-way interactions plus three-way interaction | 1680.65 | 1689.93 |
|  | Three two-way interactions | 1677.65 | 1686.93 |
|  | Clustering × Round and Bubble × Round | 1676.78 | 1686.07 |
|  | Clustering × Bubbles and Bubble × Round | 1677.13 | 1686.42 |
|  | Clustering × Bubble and Clustering × Round | 1679.71 | 1689.00 |
|  | Only Clustering × Bubble | 1679.25 | 1688.54 |
|  | Only Clustering × Round | 1678.85 | 1688.14 |
|  | Only Bubble × Round | 1676.27 | 1685.56 |
| Majority | Three two-way interactions plus three-way interaction | 3347.77 | 3358.18 |
|  | Three two-way interactions | 3345.98 | 3356.39 |
|  | Clustering × Round and Bubble × Round | 3344.87 | 3355.29 |
|  | Clustering × Bubble and Bubble × Round | 3341.78 | 3352.20 |
|  | Clustering × Bubble and Clustering × Round | 3345.08 | 3355.49 |
|  | Only Clustering × Bubble | 3340.89 | 3351.30 |
|  | Only Clustering × Round | 3343.98 | 3354.39 |
|  | Only Bubble × Round | 3340.68 | 3351.10 |

**Part IV. Sensitivity Analysis of the Minority Influence Results**

We defined minority members as seven participants whose opinions deviated from the median initial opinion of the entire group and at the same time were close to each other (see Table 1) and the majority as the remaining participants. With this classification, it is possible that some majority members held opinions closer to the median initial opinion of the minority members than to that of the majority members, making these participants more “minority friendly.” This raises an important question: When we observed that the clustering manipulation could increase the minority influence on majority members, as shown in Figure 5 and Table 3, was it driven by the minority-friendly majority members only? To address this question, we ran the same mixed-effect model as reported in Table 3 but excluding the minority-friendly majority members, whom we quantified as members whose initial opinions were closer to the initial median opinion of the minority members than to that of the majority members. As a result, 104 data points were excluded (out of 1,034 all data points), and the results of the model with the remaining data points are shown in Table S3. The main effect of clustering and the interaction effect between filter bubble and round were significant, consistent with the results with data points from all majority members.

**Table S3.** The Mixed-Effects Model Results on the Opinion Leaning Index of the Majority Members Excluding Those Minority-Friendly Members

| Fixed effects | | | | Standardized regression coefficients | | | | | |
| --- | --- | --- | --- | --- | --- | --- | --- | --- | --- |
| Variable | *df* | *F* | *p* | *β* | *SEM* | *df* | *t* | *p* | 95%CI |
| Clustering | 440.00 | 4.592 | .033 | .053 | 0.025 | 440.00 | 2.143 | .033 | [.004, .103] |
| Bubble | 978.28 | 3.165 | .076 | .057 | 0.032 | 978.28 | 1.779 | .076 | [−.006, .119] |
| Round | 884.00 | 0.805 | .370 | .008 | 0.007 | 884.00 | 1.158 | .247 | [−.006, .022] |
| Bubble × Round | 884.00 | 6.563 | .011 | −.025 | 0.010 | 884.00 | −2.562 | .011 | [−.045, −.006] |

*Note.* Bubble = filter bubble; SEM = standard error of the mean; CI = confidence interval.

**Part V. Gender Differences in Opinion**

Due to the overrepresentation of female participants in our study (370 out of 456), we examined the gender differences in opinion. As shown in Table S4, there was no gender difference in each round for Q1. For Q2, gender differences were also nonsignificant except for Round 3. For Q3, there were indeed gender differences in all rounds. However, the effect sizes of the differences were all not large (i.e., lower than 0.30), and the actual opinion differences were also small (i.e., less than 5 points in a 99-point scale).

**Tabel S4.** Gender Differences in Opinion

| Question | Round | Mean_male_ | SE_male_ | Mean_female_ | SE_female_ | *t* | *p* | Cohen’s *d* |
| --- | --- | --- | --- | --- | --- | --- | --- | --- |
| Q1 | 0 | 8.59 | 2.079 | 6.51 | 0.832 | 0.927 | .355 | 0.096 |
| Q1 | 1 | 6.91 | 1.739 | 5.93 | 0.670 | 0.527 | .599 | 0.055 |
| Q1 | 2 | 6.96 | 1.507 | 5.77 | 0.596 | 0.733 | .464 | 0.076 |
| Q1 | 3 | 6.97 | 1.447 | 6.20 | 0.565 | 0.496 | .621 | 0.052 |
| Q2 | 0 | 8.12 | 2.119 | 5.86 | 0.799 | 0.996 | .321 | 0.105 |
| Q2 | 1 | 8.60 | 1.635 | 6.02 | 0.641 | 1.469 | .144 | 0.153 |
| Q2 | 2 | 8.57 | 1.620 | 6.07 | 0.581 | 1.453 | .148 | 0.155 |
| Q2 | 3 | 9.79 | 1.545 | 6.47 | 0.530 | 2.036 | .044 | 0.219 |
| Q3 | 0 | 28.69 | 1.699 | 24.18 | 0.809 | 2.396 | .018 | 0.236 |
| Q3 | 1 | 30.96 | 1.319 | 27.42 | 0.633 | 2.419 | .017 | 0.237 |
| Q3 | 2 | 32.70 | 1.208 | 29.08 | 0.576 | 2.706 | .007 | 0.266 |
| Q3 | 3 | 34.11 | 1.180 | 30.20 | 0.548 | 3.006 | .003 | 0.298 |

**Part VI. Proportion of Flipped Participants**

Table S5 reports the proportion of participants who *flipped* their opinions among the minority and the majority members in each experimental condition. A participant was deemed to have flipped their opinion if their opinion in Round 0 was closer to the initial median of the minority (or majority) opinions, but in Round 3 had become closer to the initial median of the majority (or minority) opinions. The results indicate that the clustering manipulation reduced the proportion of flipped minority members, whereas filter bubble reduced the proportion for both minority and majority members with a stronger effect on the minority.

Flipping captures a directional reversal of member opinions, whereas the opinion leaning index that we examined in the main text reflects a more subtle, continuous shift in opinion tendency. Taken together, these two measures suggest that our manipulations effectively shifted the majority’s opinion tendencies, even if a complete opinion reversal was relatively rare and may require stronger or more prolonged interventions for the majority members.

**Table S5.** Proportion of Flipped Participants

| Filter bubble | Clustering | Flipping proportion of minority members | Flipped proportion of majority members |
| --- | --- | --- | --- |
| No | No | 40.48% | 6.35% |
| No | Yes | 27.38% | 6.02% |
| Yes | No | 33.73% | 3.77% |
| Yes | Yes | 21.69% | 5.22% |
